# Supplementary material for: Mastering morphology of non-fullerene acceptors towards long-term stable organic solar cells
Source: Nat Commun. 2023 May 10;14:2688. doi: 10.1038/s41467-023-38306-x (PMC10172308; doi:10.1038/s41467-023-38306-x)
Supplement: Supplementary file 1 — Supplementary Information [file 41467_2023_38306_MOESM1_ESM.pdf]

SUPPLEMENTARY INFORMATION

Mastering Morphology of Non-fullerene Acceptors towards Long-term Stable Organic Solar Cells

Kang An,[1] Wenkai Zhong,[1,2] Feng Peng,[3] Wanyuan Deng,[1] Ying Shang,[1,4] Huilei Quan,[1] Hong Qiu,[1] Cheng Wang,[5] Feng Liu,[2] Hongbin Wu,[1] Ning Li,[1,4*] Fei Huang,[1,4*] and Lei Ying[1,*]

[1]Institute of Polymer Optoelectronic Materials and Devices, State Key Laboratory of Luminescent Materials and Devices, South China University of Technology, Guangzhou, 510640, China. [2]Frontiers Science Center for Transformative Molecules, Center of Hydrogen Science, and School of Chemistry and Chemical Engineering, Shanghai Jiao Tong University, Shanghai, 200240, China. [3]South China Institute of Collaborative Innovation, Dongguan, 523808, China. [4]Pazhou Lab, Guangzhou, 510320, China. [5]Advanced Light Source Lawrence Berkeley National Laboratory Berkeley, CA 94720, USA. These authors contributed equally: Kang An, Wenkai Zhong and Feng Peng.

[*]E-mail: ningli2022@scut.edu.cn; msfhuang@scut.edu.cn; msleiying@scut.edu.cn

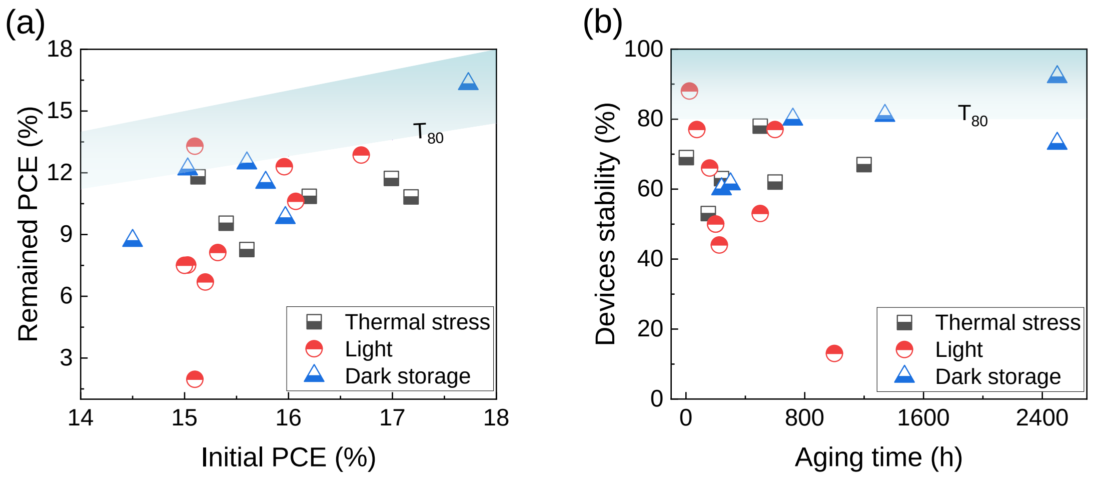

Supplementary Fig. 1 | Stability statistics. (a) Stability statistics of organic solar cells (OSCs) based on Y6 and its derivatives under thermal stress, light and dark storage aging conditions reported by previous literatures. (b) The aging time versus remained PCE percentage in corresponding OSCs. The gradient filled zones represent the lifetime from T_{80} to T_{100}.

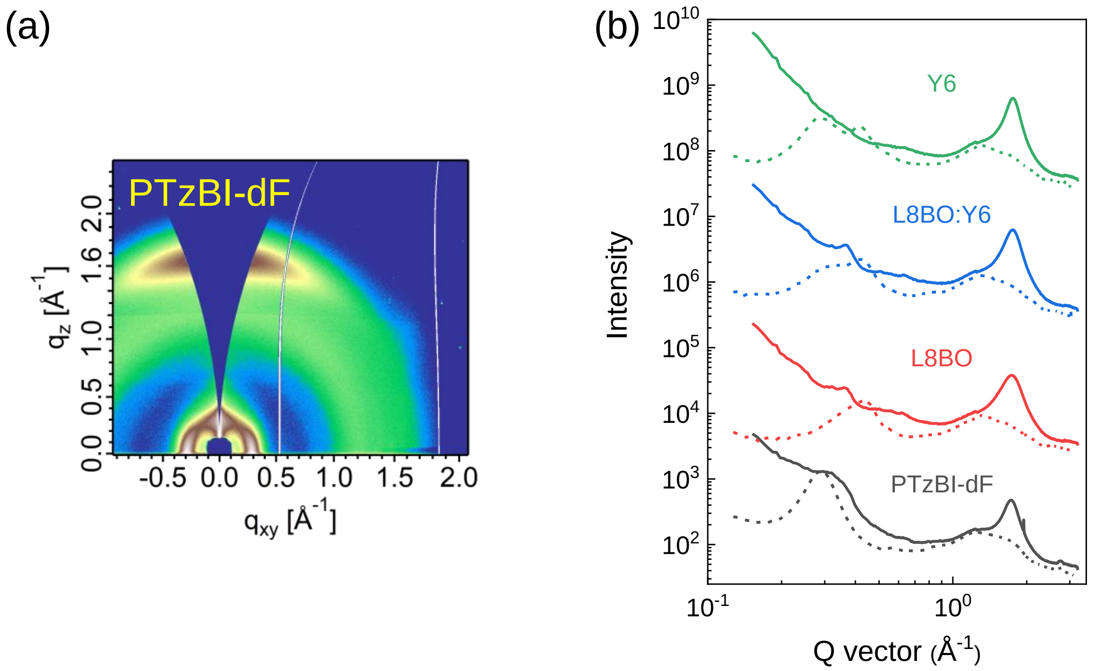

Supplementary Fig. 2 | Molecular packing. Grazing incidence wide-angle X-ray scattering (GIWAXS) 2D patterns (a) and sector line-cuts (b) (solid lines: OOP; dotted lines: IP) for the PTzBI-dF, L8BO, L8BO:Y6 (0.7:0.5) and Y6 films.

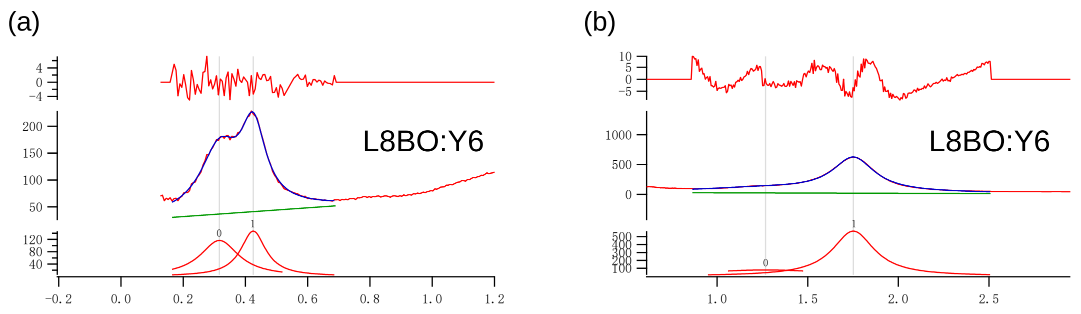

Supplementary Fig. 3 | Molecular packing. The (a) in-plane and (b) out-of-plane line cuts for the L8BO:Y6 blend and the demonstration of the curve fitting.

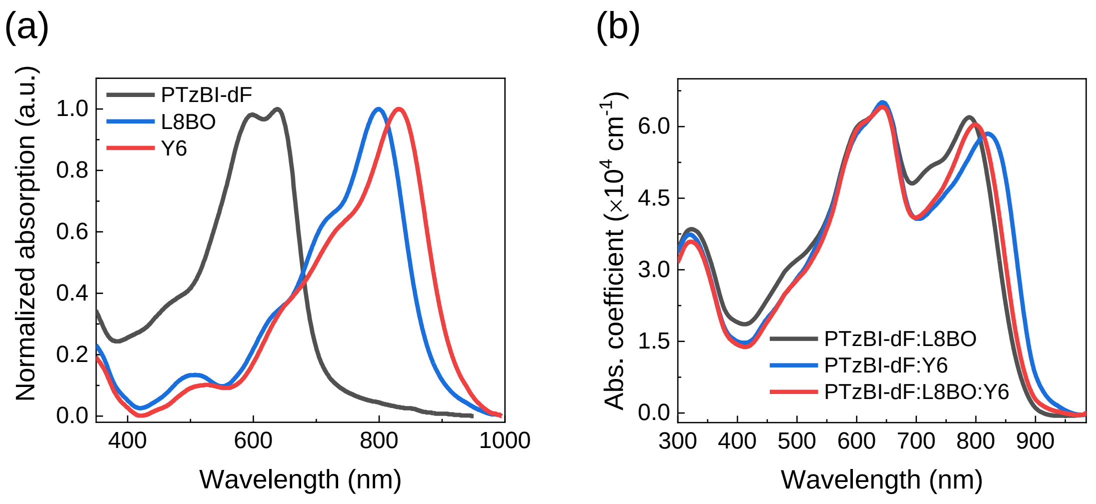

Supplementary Fig. 4 | Absorption spectra. (a) Normalized absorption spectra for PTzBI-dF, L8BO and Y6 neat films. (b) Absorption coefficients.

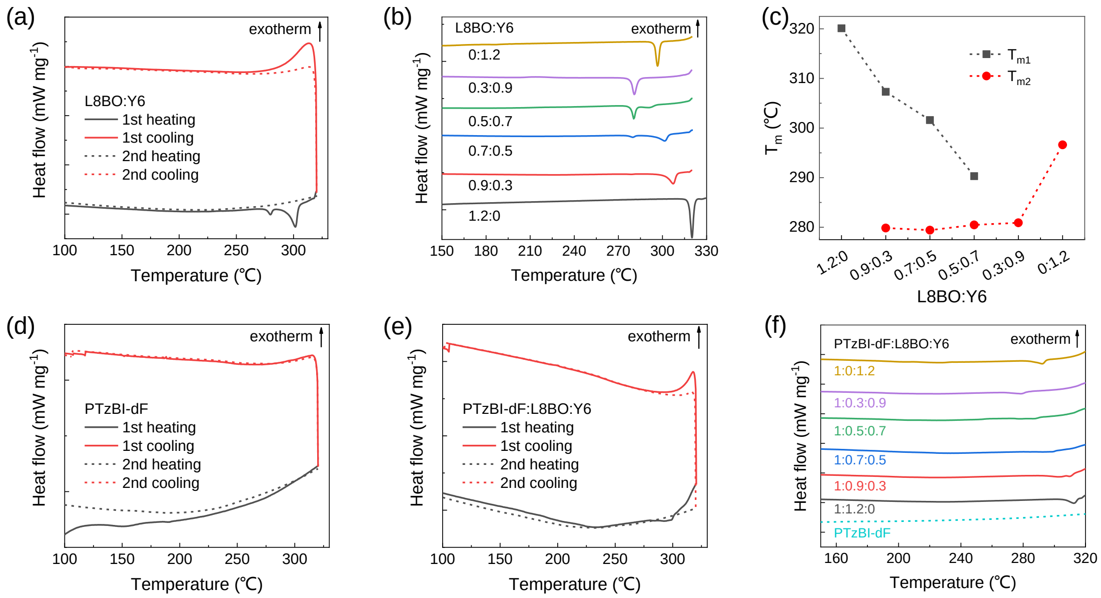

Supplementary Fig. 5 | Thermal behavior. (a) Differential scanning calorimetry (DSC) first and second heating and cooling curves from L8BO:Y6 blends (0.7:0.5). (b) DSC first heating curves for L8BO:Y6 blends with various ratios. (c) The melting points (T_m) for L8BO:Y6 blends; T_{m1} indicate L8BO-related melting peak; T_{m2} indicate Y6-related melting peak. DSC first and second heating and cooling curves from (d) PTzBI-dF neat and (e) PTzBI-dF:L8BO:Y6 blends. (f) DSC first heating curves for PTzBI-dF neat films and PTzBI-dF:L8BO:Y6 blends with various ratios.

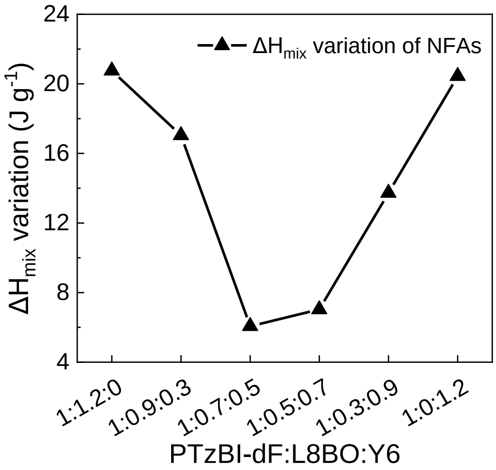

Supplementary Fig. 6 | Thermal behavior. The ΔH_{mix} variation obtained from the DSC of PTzBI-dF:L8BO:Y6 blends via deducting the weight of the polymer donor PTzBI-dF.

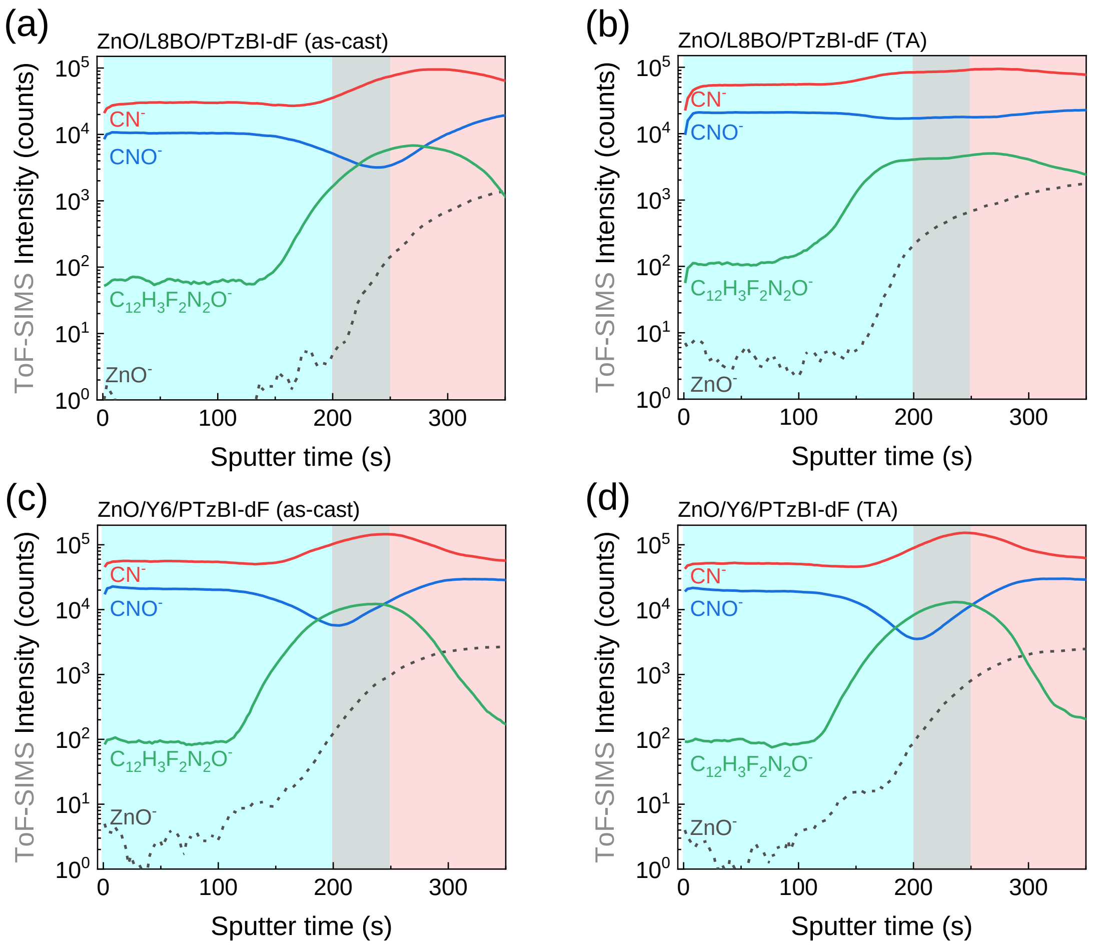

Supplementary Fig. 7 | TOF-SIMS. TOF-SIMS of PTzBI-dF/L8BO (a, b) and PTzBI-dF/Y6 (c, d) bilayer films under the as-cast and post thermal annealing (TA) conditions. The bilayer films were prepared on ZnO/Si wafer substrates.

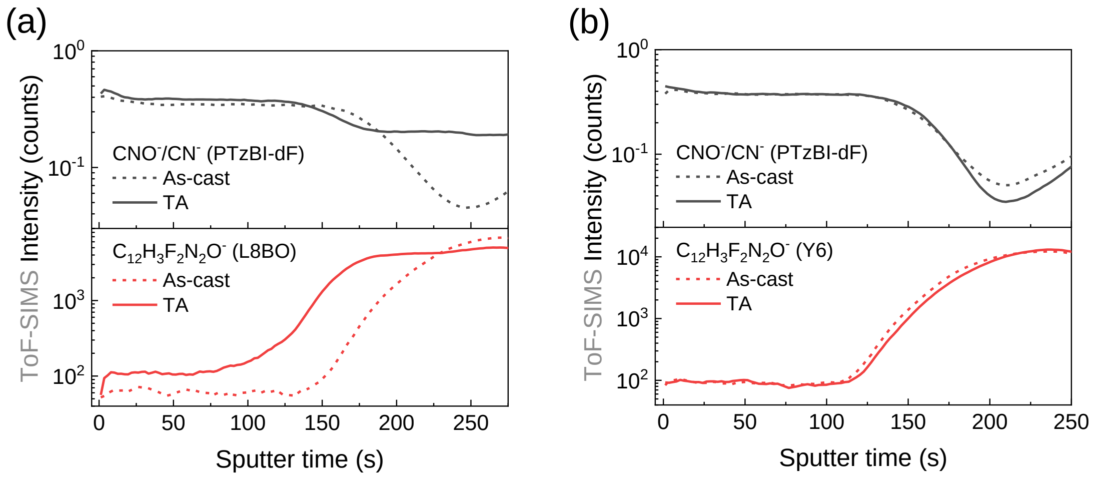

Supplementary Fig. 8 | TOF-SIMS. Depth profiles for as-cast and TA bilayer structures of (a) ZnO/L8BO/PTzBI-dF and (b) and ZnO/Y6/PTzBI-dF, respectively. TOF-SIMS results in Supplementary Table 4 reveals that both CNO^- and CN^- signals can be generated by PTzBI-dF and NFAs. In the case of PTzBI-dF, the intensity of CNO^- is higher than that of CN^-, whereas for NFAs, the opposite is observed, with the intensity of CNO^- being lower than that of CN-. As a result, the CNO^-/CN^- intensity ratio is utilized to track the distribution of PTzBI-dF, while for the NFAs, the end group segment of $C_{12}H_3F_2N_2O^-$ is used as a label.

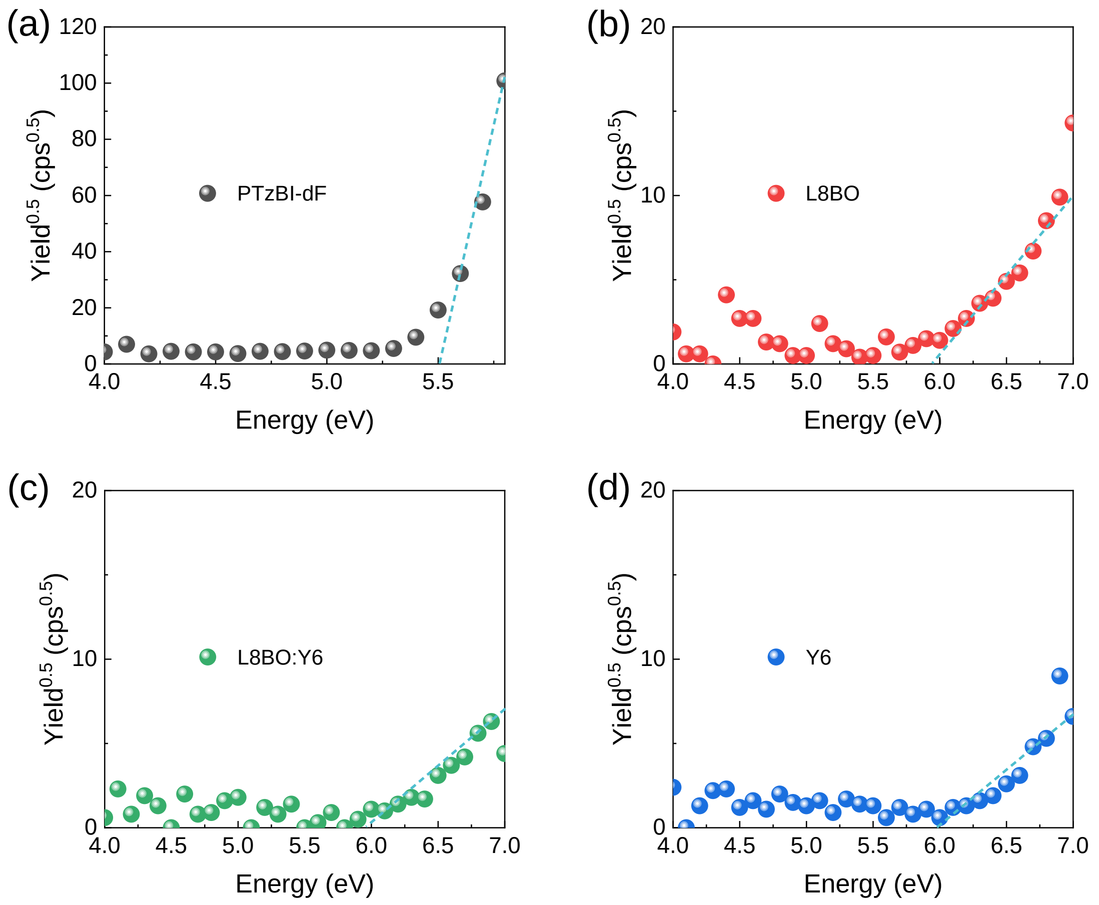

Supplementary Fig. 9 | Energy levels. Photoemission yield spectroscopy for (a) PTzBI-dF, (b) L8BO, (c) L8BO:Y6 and (d) Y6 films.

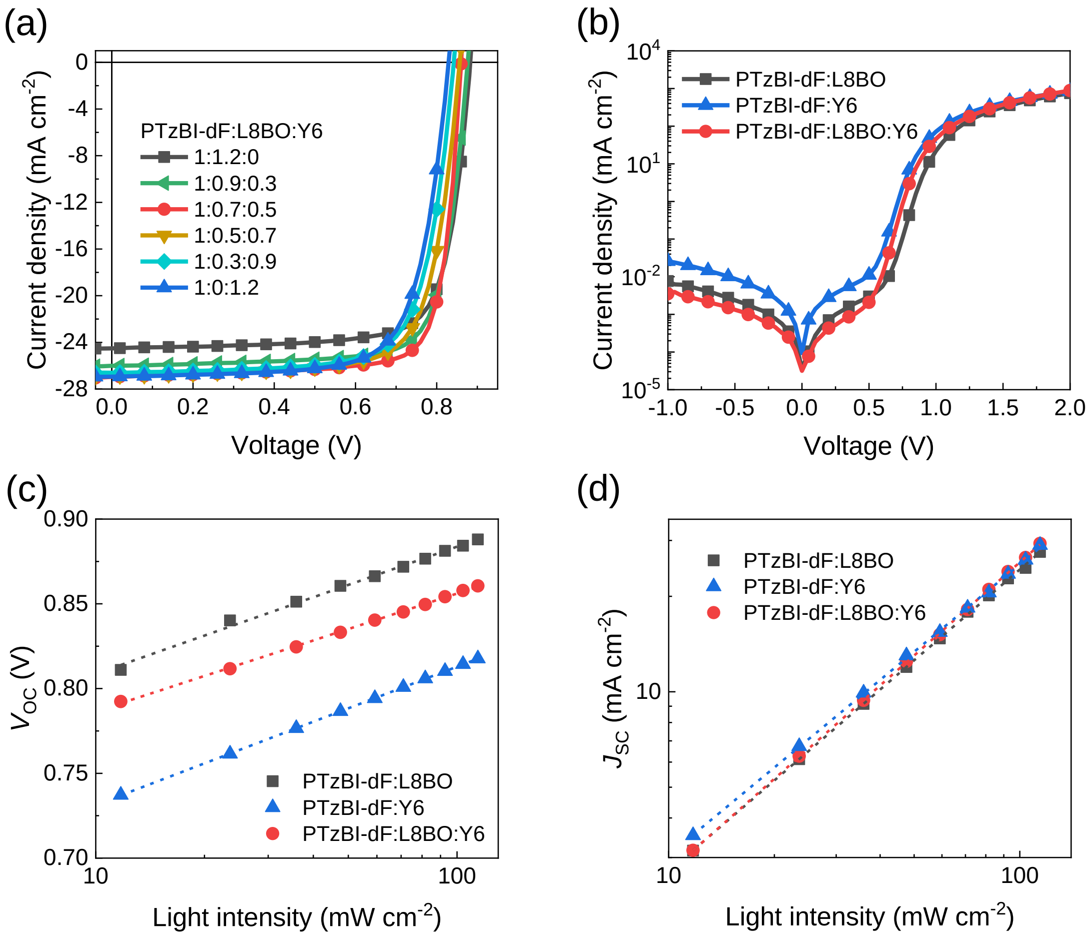

Supplementary Fig. 10 | Photovoltaic performance. (a) The current density–voltage ($J-V$) characteristics of PTzBI-dF based OSCs with various L8BO:Y6 blending ratios under simulated AM 1.5G irradiation (100 mW cm^{-2}). (b) The $J-V$ characteristics under dark. The dependence of (c) V_{OC} and (d) J_{SC} on light intensity (P_{light}) measurements for PTzBI-dF:L8BO, PTzBI-dF:Y6 and PTzBI-dF:L8BO:Y6 based OSCs.

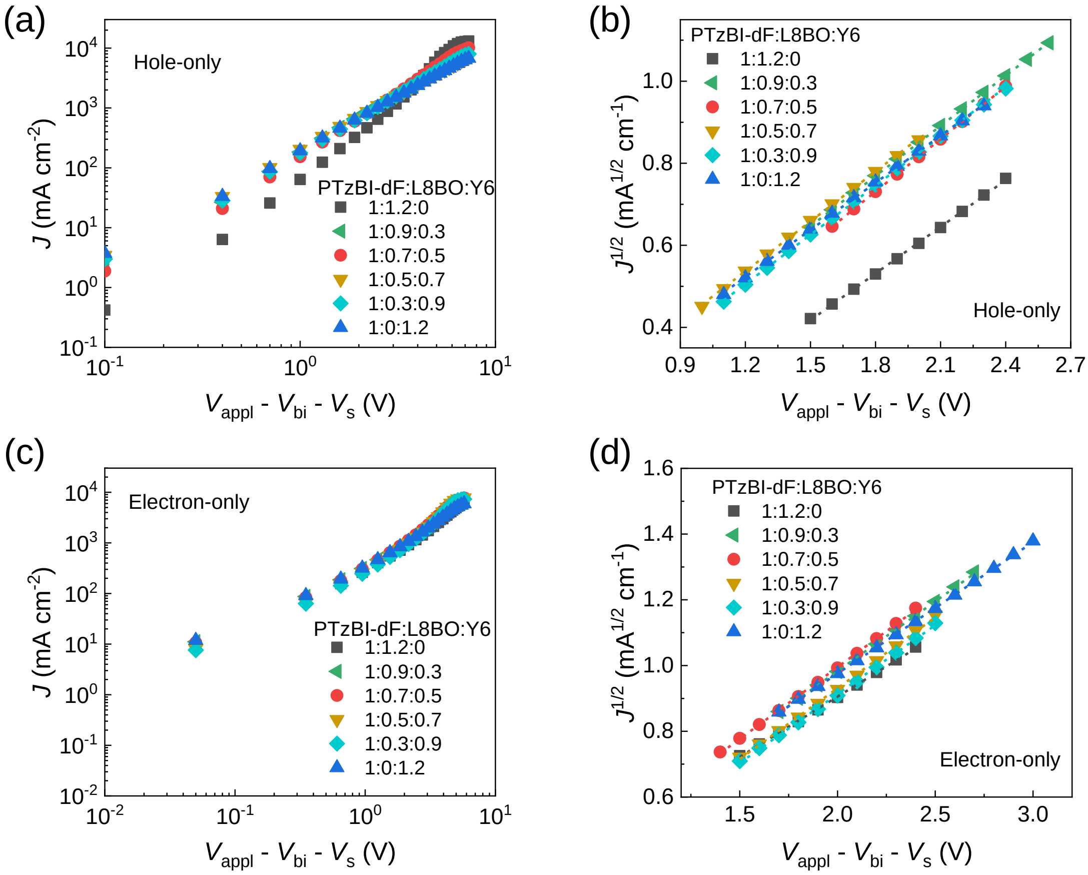

Supplementary Fig. 11 | Charge mobilities. $J - V$ (a, c) and $J^{1/2} - V$ (b, d) characteristics for hole-only and electron-only devices based on PTzBI-dF:L8BO:Y6 systems with various L8BO:Y6 ratios.

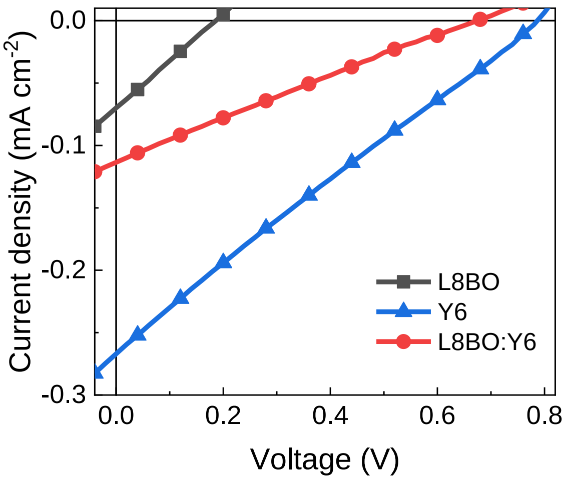

Supplementary Fig. 12 | Charge transfer. The J–V characteristics of specific devices based on L8BO:Y6 with different blending ratios under simulated AM 1.5G irradiation (100 mW cm^{-2}).

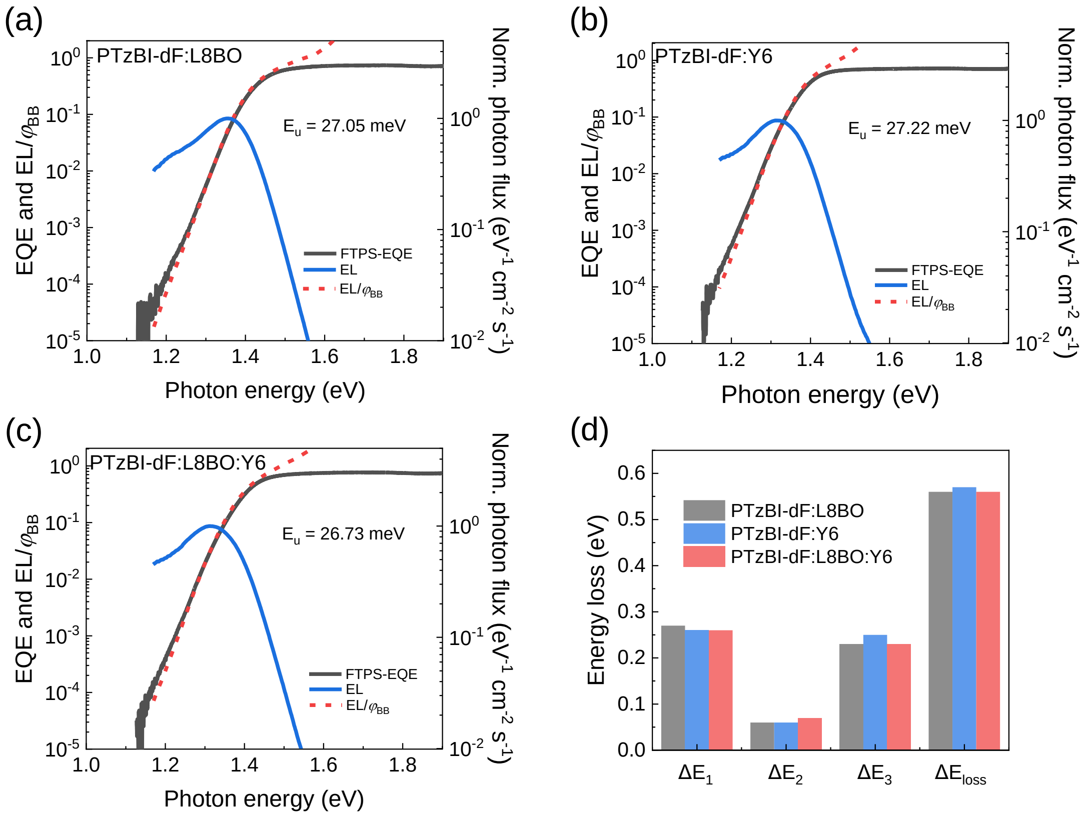

Supplementary Fig. 13 | Energy loss. EQE calculated by FTPS (FTPS-EQE), normalized EL and EL/φ_{BB} as a function of photon energy for (a) PTzBI-dF:L8BO, (b) PTzBI-dF:Y6 and (c) PTzBI-dF:L8BO:Y6 based OSCs. (d) The summary of energy losses.

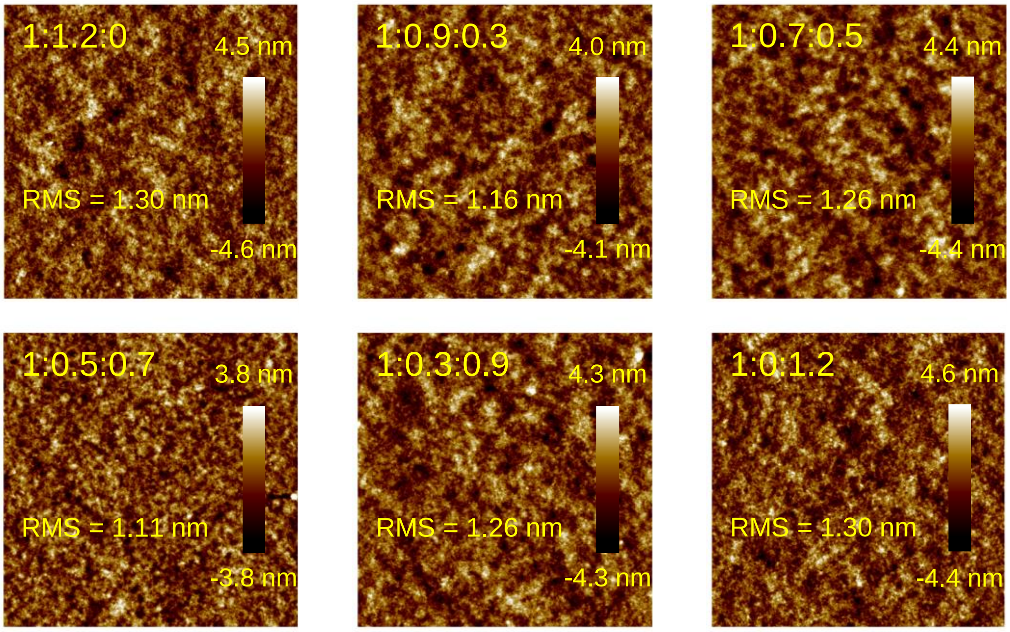

Supplementary Fig. 14 | Morphology. AFM height images (5 μm×5 μm scale) for PTzBI-dF:L8BO:Y6 based blends with various L8BO:Y6 blending ratios.

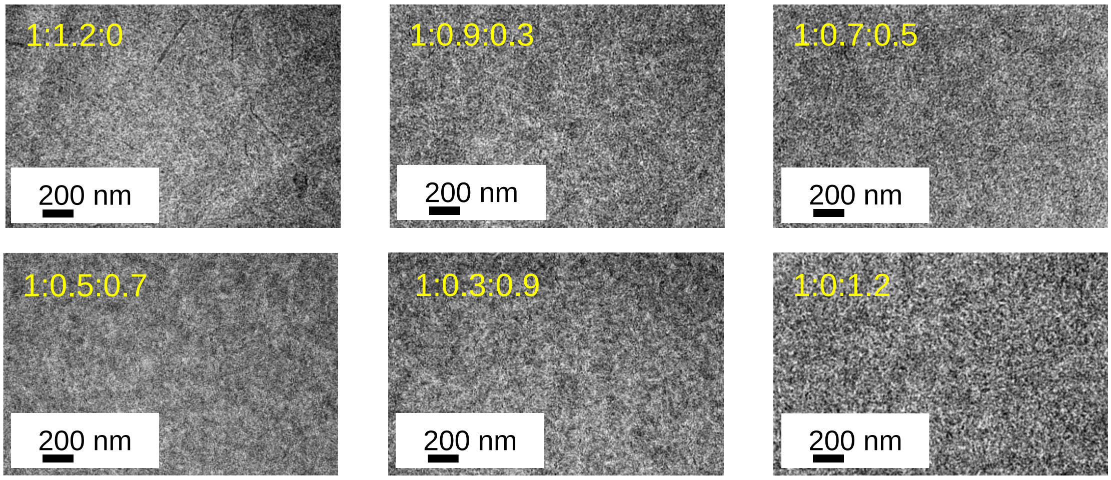

Supplementary Fig. 15 | Morphology. TEM images for PTzBI-dF:L8BO:Y6 based blends with various L8BO:Y6 blending ratios.

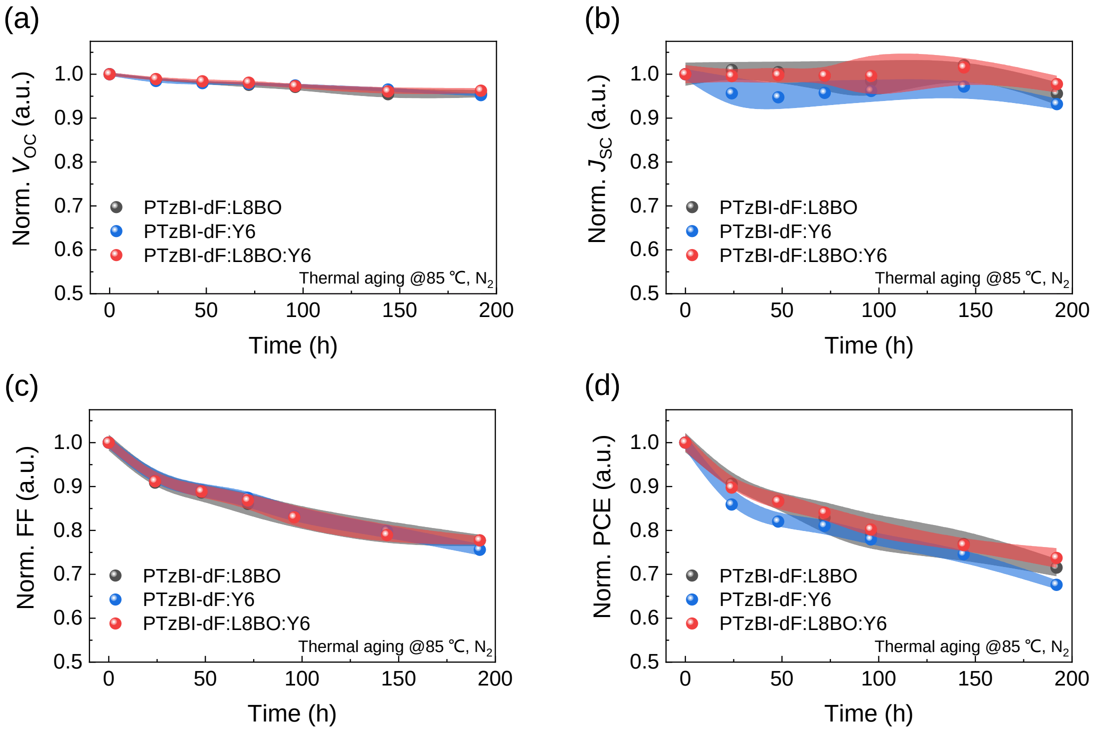

Supplementary Fig. 16 | Thermal stability. Normalized parameters of (a) V_{OC}, (b) J_{SC}, (c) FF and (d) PCE for PTzBI-dF:L8BO, PTzBI-dF:Y6, PTzBI-dF:L8BO:Y6 devices with conventional structure under continuous 85 °C ageing in a dry nitrogen atmosphere without encapsulation.

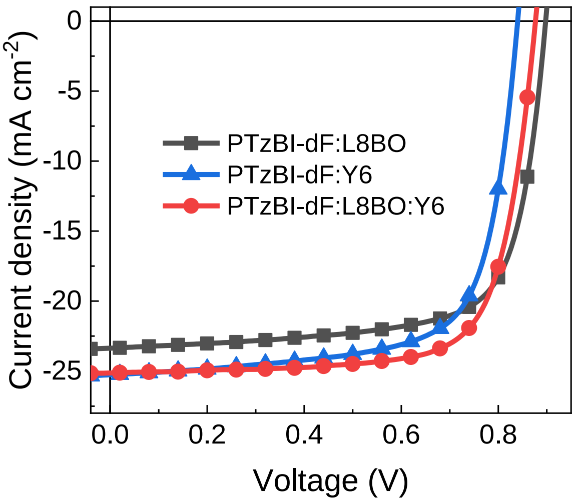

Supplementary Fig. 17 | Photovoltaic performance. The *J*–*V* characteristics of PTzBI-dF:L8BO, PTzBI-dF:Y6 and PTzBI-dF:L8BO:Y6 inverted devices under simulated AM 1.5G irradiation (100 mW cm^{-2}).

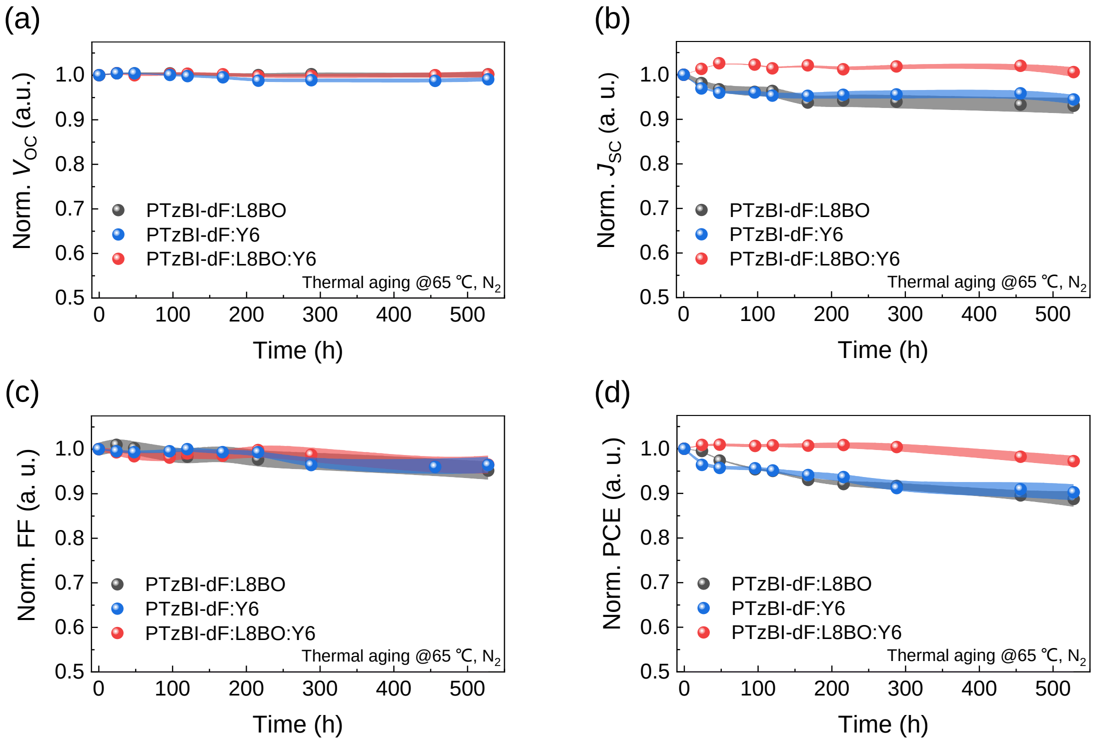

Supplementary Fig. 18 | Thermal stability. Normalized parameters of (a) V_{OC}, (b) J_{SC}, (c) FF and (d) PCE for PTzBI-dF:L8BO, PTzBI-dF:Y6, PTzBI-dF:L8BO:Y6 devices with inverted structure under continuous 65 ℃ ageing in a dry nitrogen atmosphere without encapsulation.

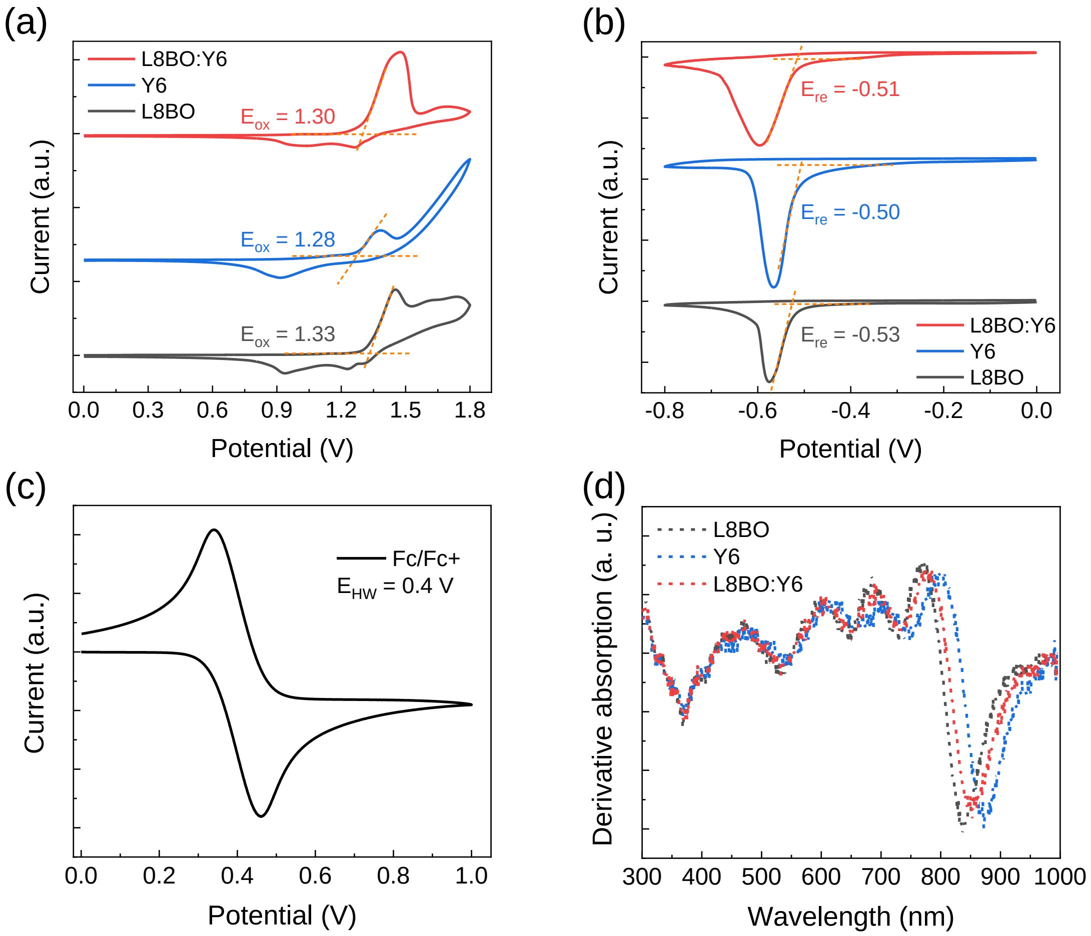

Supplementary Fig. 19 | Cyclic voltammetry. (a) Ionization energy and (b) electron affinity of L8BO, Y6 and L8BO:Y6 acceptors determined by cyclic voltammetry (CV). (c) Half-wave potential of ferrocene reference. (d) Derivative of corresponding absorption spectra.

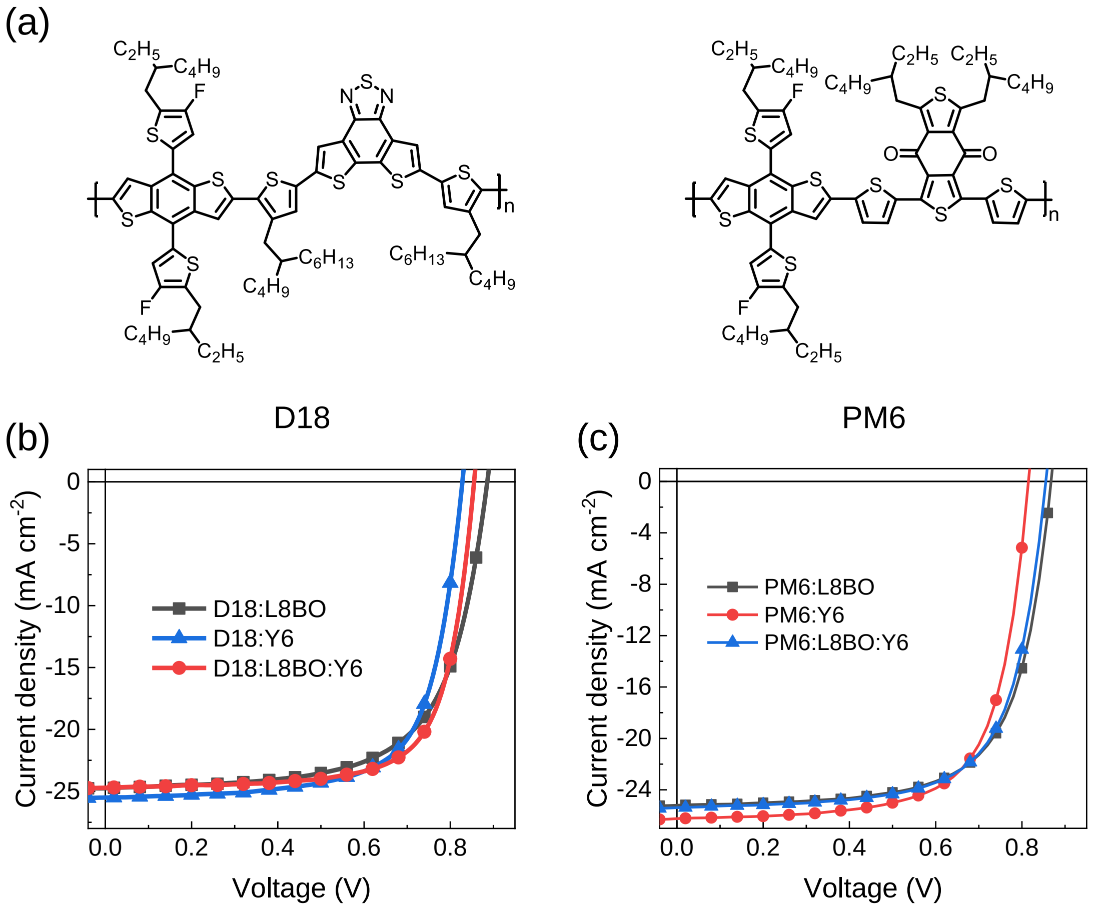

Supplementary Fig. 20 | Photovoltaic performance. (a) The chemical structure of D18 and PM6 polymer donors. (b, c) The *J*-*V* curves of D18 and PM6 separately combined with L8BO, Y6 and L8BO:Y6 as active layers.

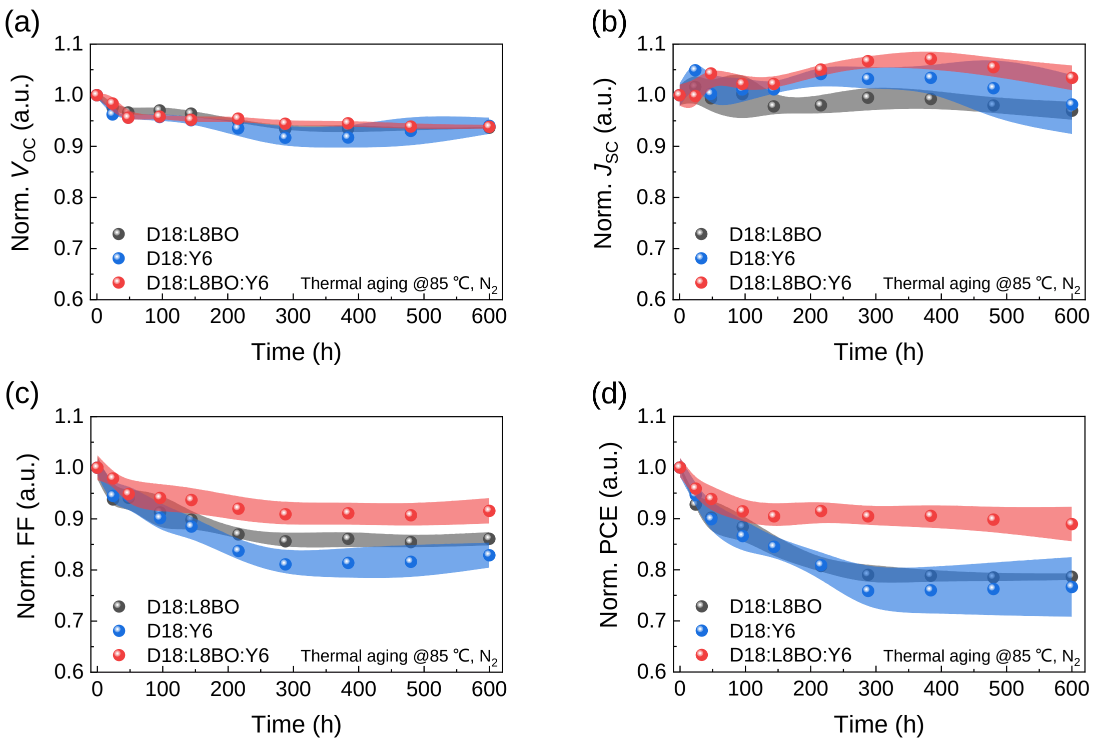

Supplementary Fig. 21 | Thermal stability. Normalized parameters of (a) V_{OC}, (b) J_{SC}, (c) FF and (d) PCE for D18:L8BO, D18:Y6 and D18:L8BO:Y6 devices under continuous 85 °C ageing in a dry nitrogen atmosphere without encapsulation.

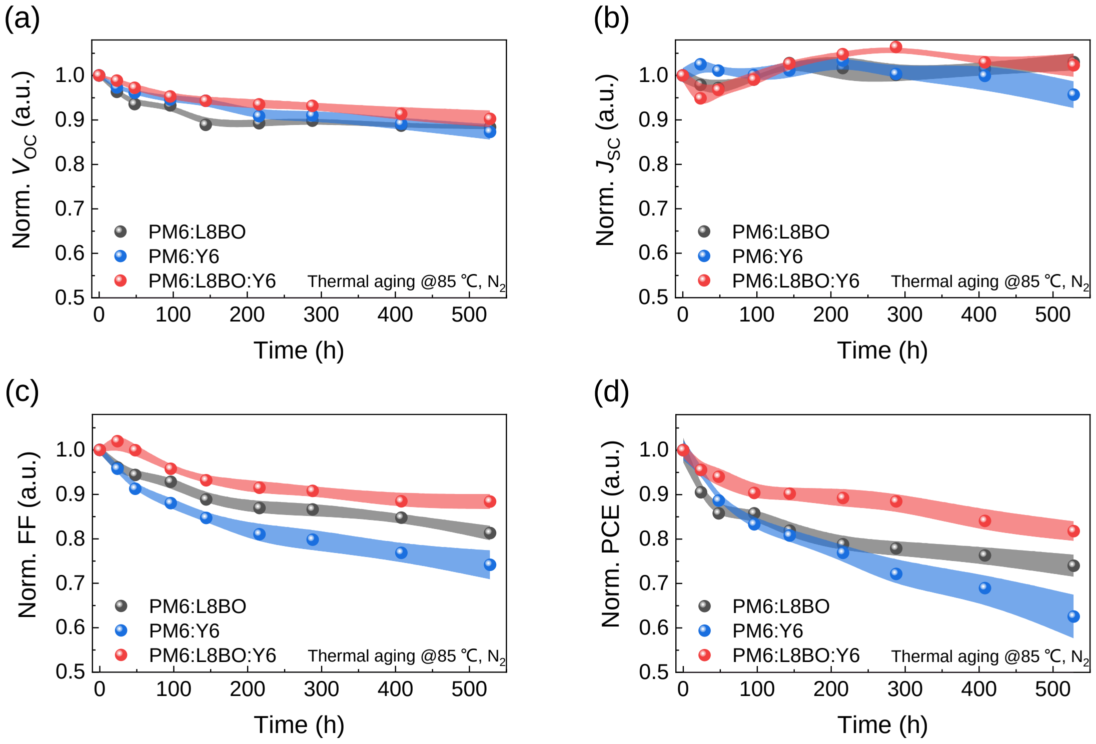

Supplementary Fig. 22 | Thermal stability. Normalized parameters of (a) V_{OC}, (b) J_{SC}, (c) FF and (d) PCE for PM6:L8BO, PM6:Y6 and PM6:L8BO:Y6 devices under continuous 85 °C ageing in a dry nitrogen atmosphere without encapsulation.

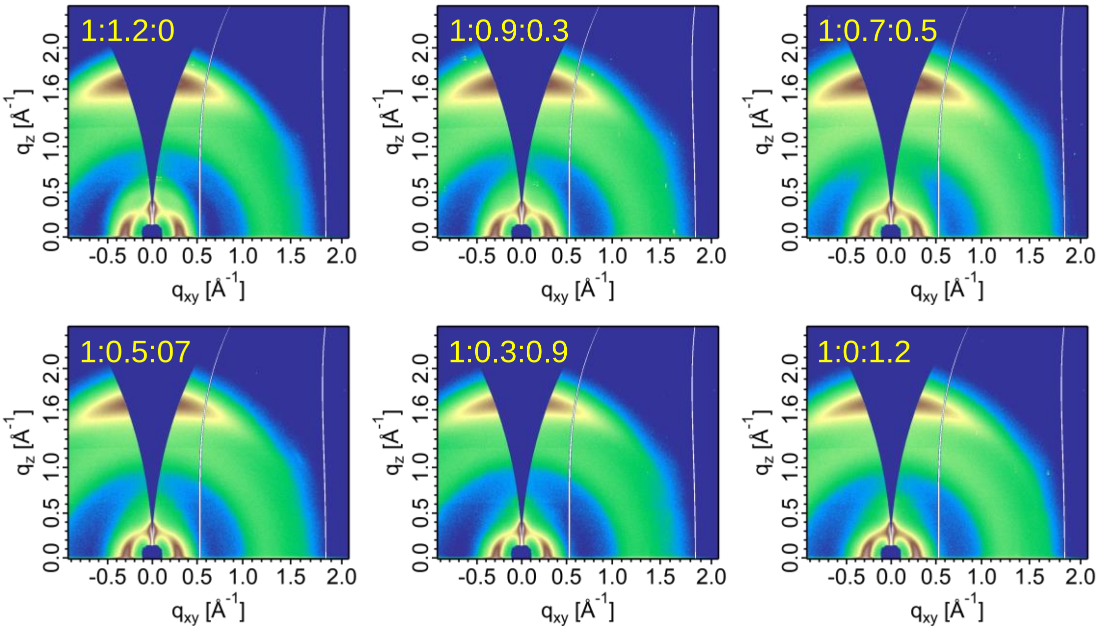

Supplementary Fig. 23 | Morphology. GIWAXS 2D patterns for fresh PTzBI-dF:L8BO:Y6 blend films with various L8BO:Y6 ratios.

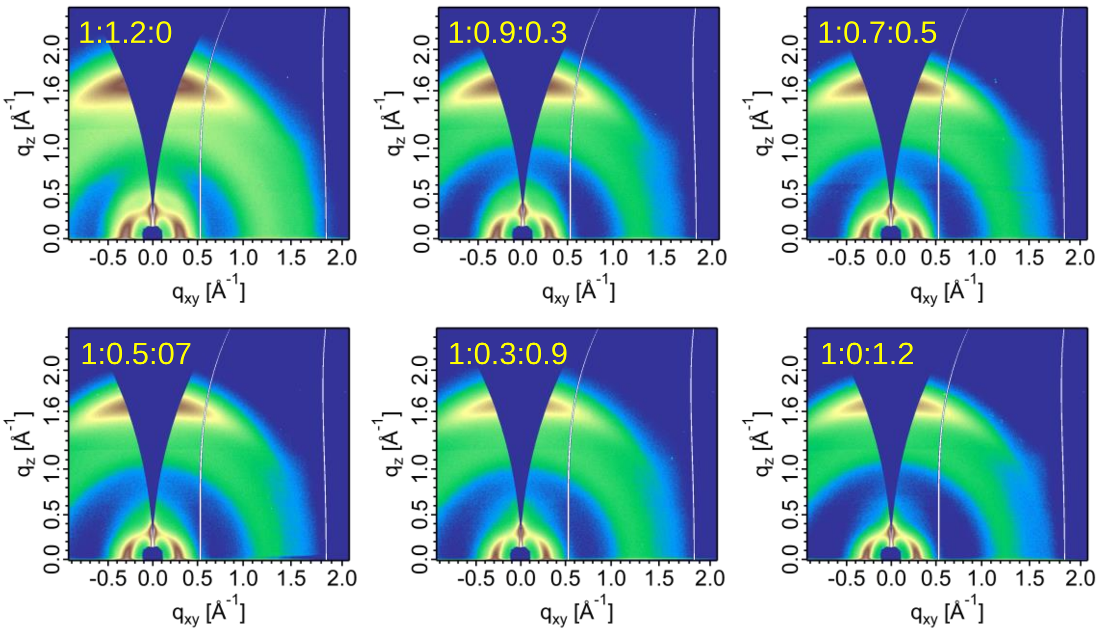

Supplementary Fig. 24 | Morphology. GIWAXS 2D patterns for aged PTzBI-dF:L8BO:Y6 blend films with various L8BO:Y6 ratios.

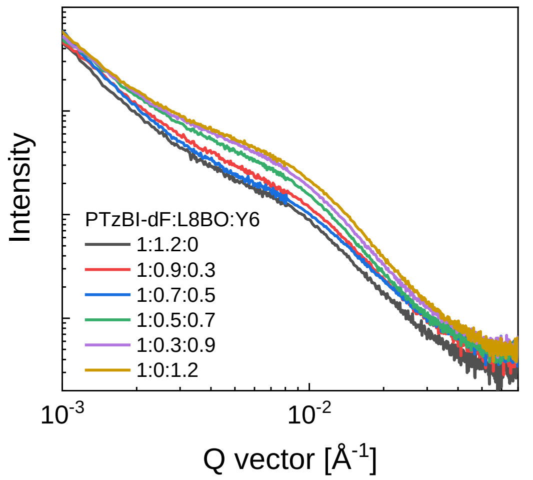

Supplementary Fig. 25 | Morphology. RSoXS averaged profiles of PTzBI-dF:L8BO:Y6 blend films with various L8BO:Y6 ratios.

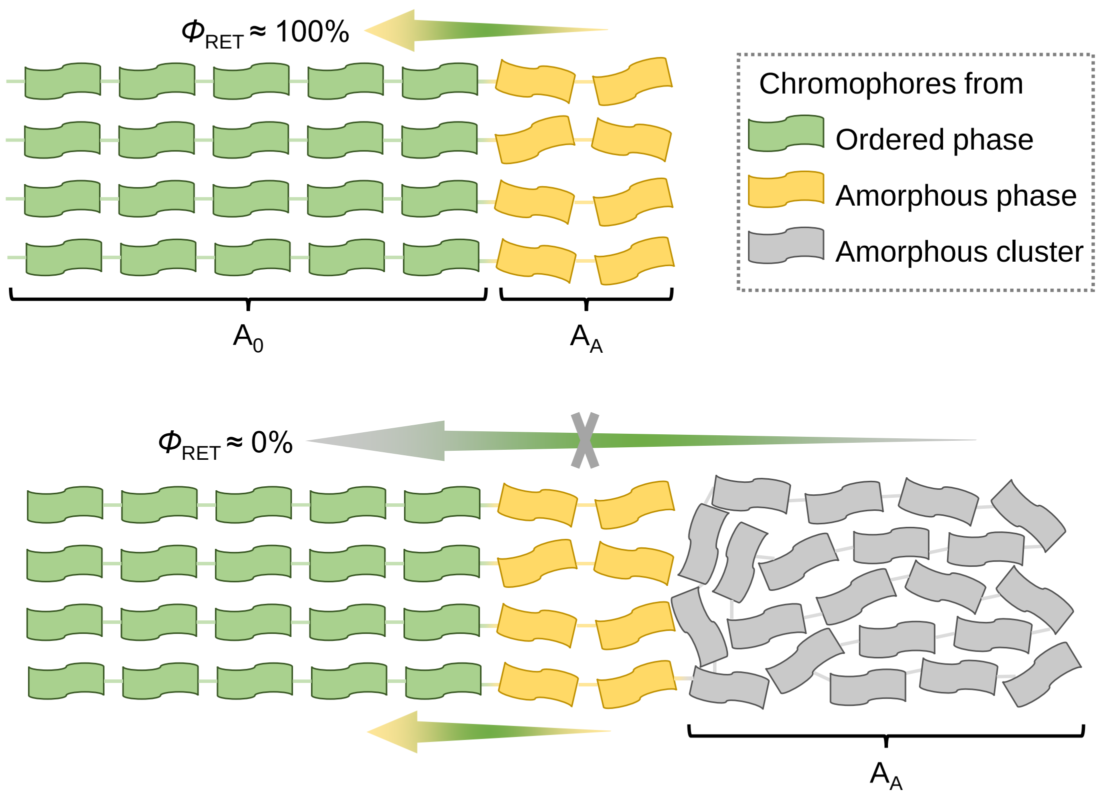

Supplementary Fig. 26 | Sketch of a disordered BHJ. Ordered phase, amorphous phase in folding region, and amorphous cluster marked by different colors.

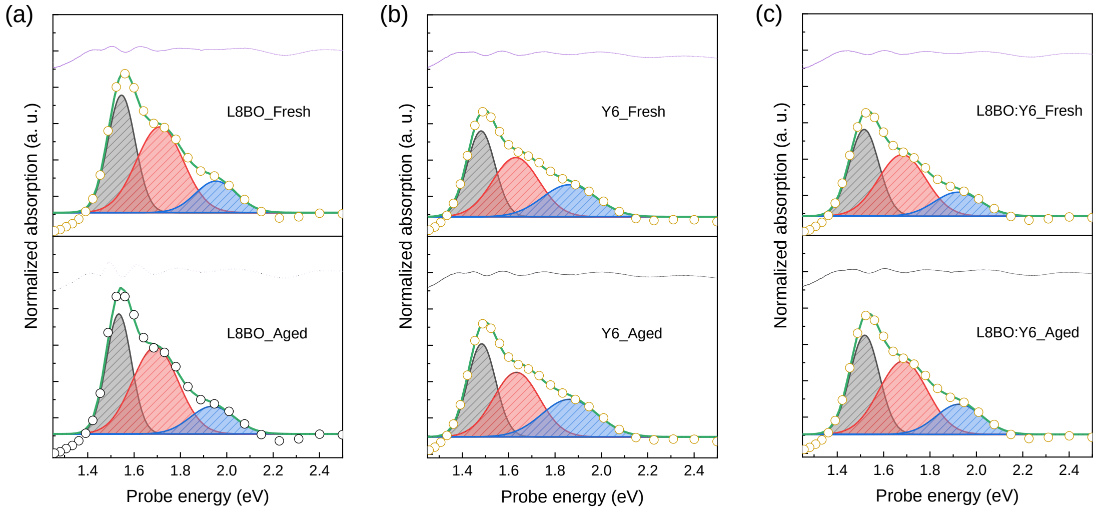

Supplementary Fig. 27 | UV-vis-NIR spectra. UV-vis-NIR spectra of fresh and aged films for (a) L8BO, (b) Y6 and (c) L8BO:Y6 blends. Hollow circle symbols are experimental data results; green lines are spectral fits obtained by superposition of electronic contributions; black shaded areas indicate ordered phase; red shaded areas indicate amorphous phase; blue shades areas indicate higher electronic transitions.

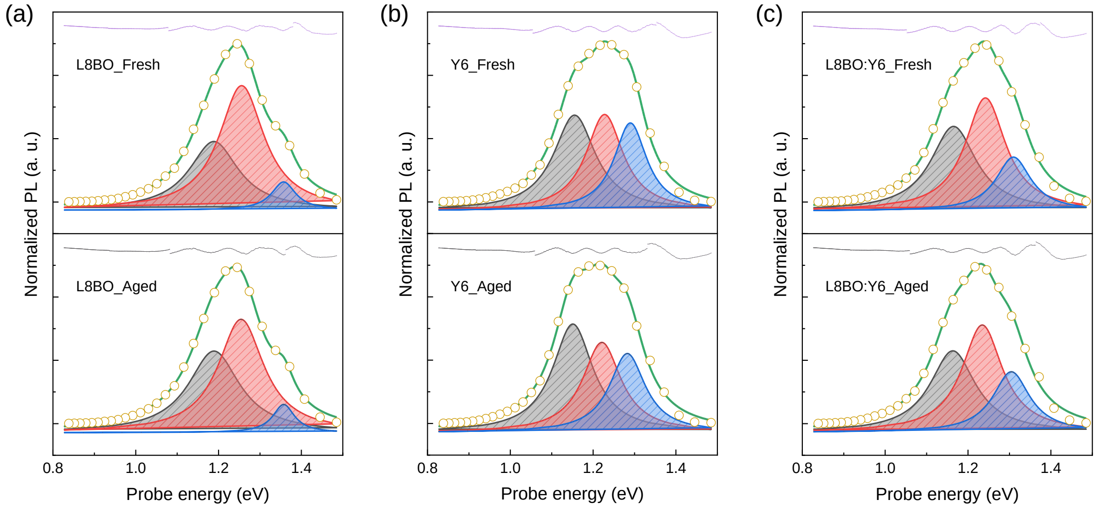

Supplementary Fig. 28 | PL profiles. PL profiles of fresh and aged films for (a) L8BO, (b) Y6 and (c) L8BO:Y6 blends. Hollow circle symbols are experimental data results; green lines are spectral fits obtained by superposition of electronic contributions; lines with shaded areas are contributions of (01) transition (black), (00) transition (red) and PL_A band (blue).

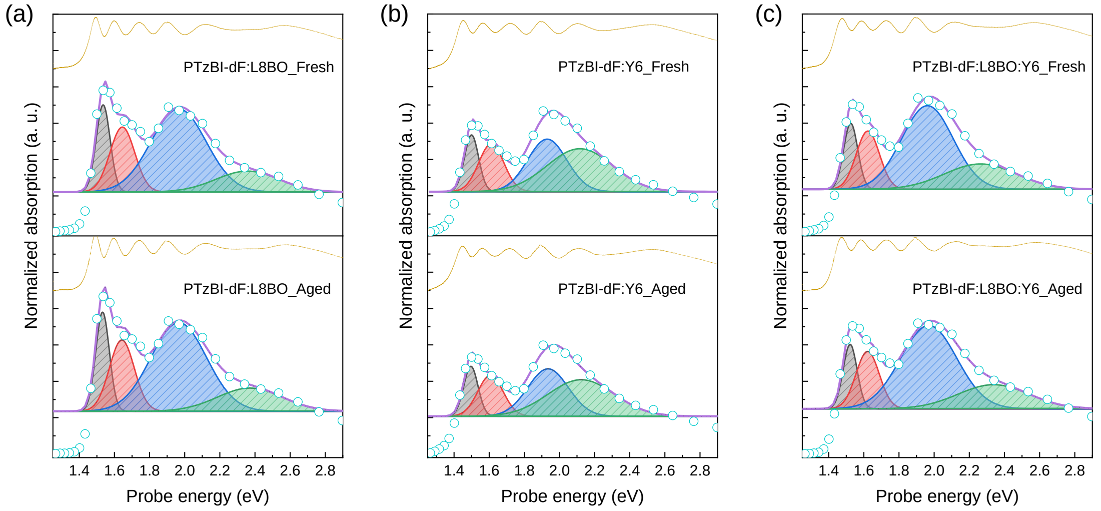

Supplementary Fig. 29 | UV-vis spectra. UV-vis-NIR spectra of fresh and aged films for (a) PTzBI-dF:L8BO, (b) PTzBI-dF:Y6 and (c) PTzBI-dF:L8BO:Y6 blends. Hollow circle symbols are experimental data results; purple lines are spectral fits obtained by superposition of electronic contributions; dashed lines of black/red and blue/green showed the contribution of the basis spectra of polymer donor and NFAs, respectively.

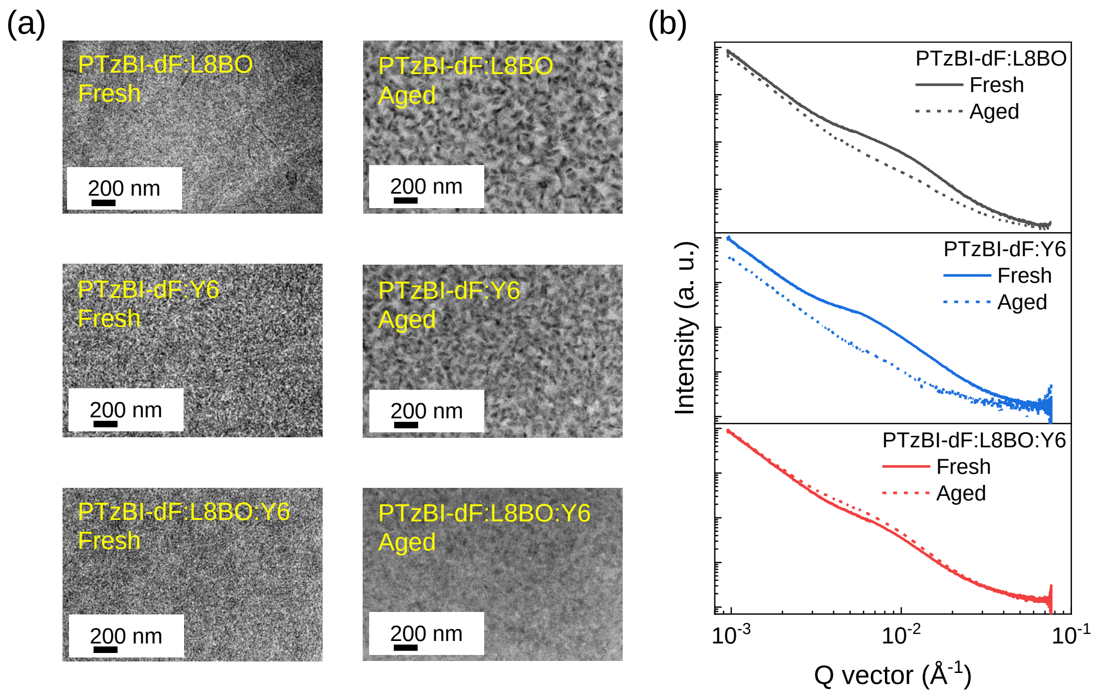

Supplementary Fig. 30 | Morphology. (a) TEM images and (b) RSoXS averaged profiles for fresh and aged PTzBI-dF:L8BO, PTzBI-dF:Y6 and PTzBI-dF:L8BO:Y6 films.

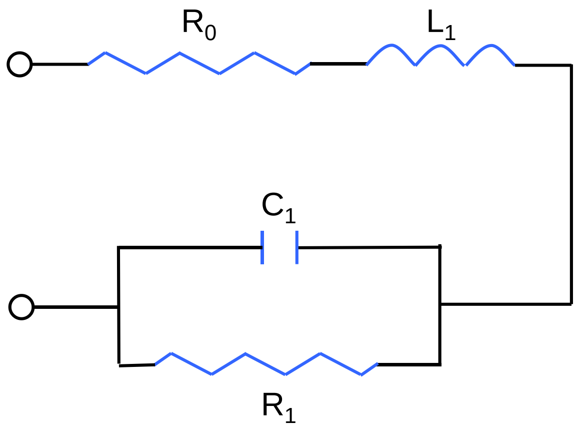

Supplementary Fig. 31 | Equivalent circuits. The equivalent circuits used in impedance spectra.

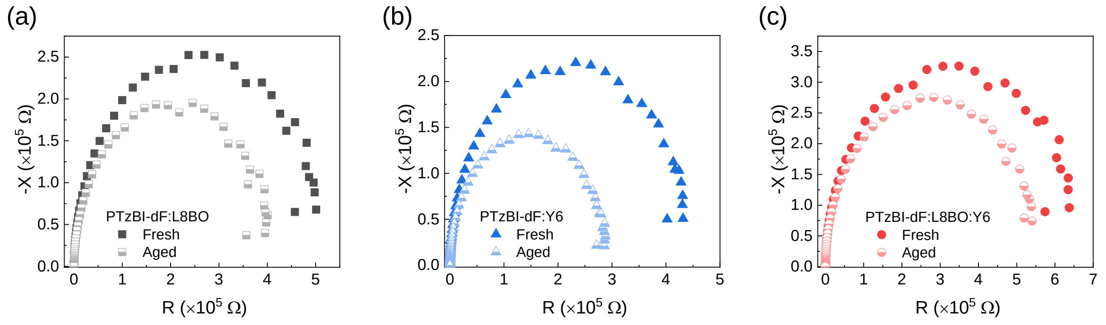

Supplementary Fig. 32 | Impedance spectra. Impedance spectra for fresh and aged (a) PTzBI-dF:L8BO, (b) PTzBI-dF:Y6 and (c) PTzBI-dF:L8BO:Y6 based devices.

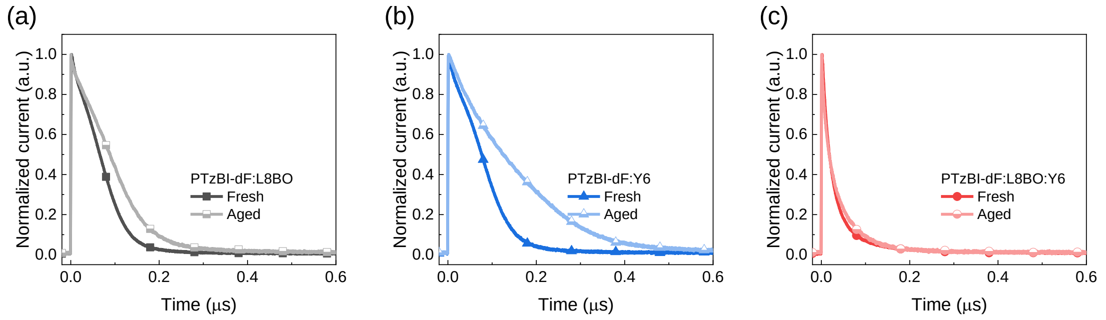

Supplementary Fig. 33 | TPC. Normalized transient current curves for fresh and aged (a) PTzBI-dF:L8BO, (b) PTzBI-dF:Y6 and (c) PTzBI-dF:L8BO:Y6 based devices.

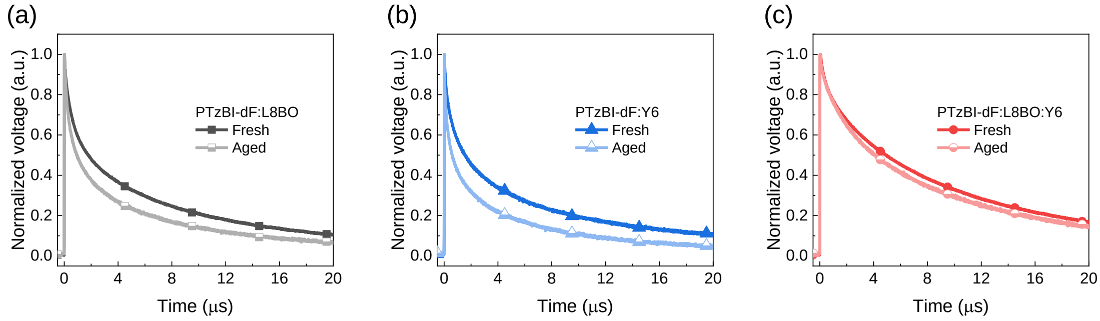

Supplementary Fig. 34 |TPV. Normalized transient photovoltage curves for fresh and aged (a) PTzBI-dF:L8BO, (b) PTzBI-dF:Y6 and (c) PTzBI-dF:L8BO:Y6 based devices.

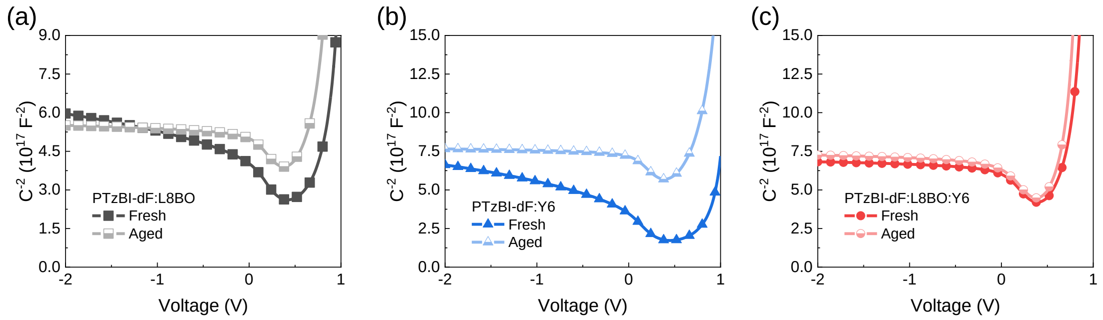

Supplementary Fig. 35 | *C-V*. C^{-2} versus applied voltage curves for fresh and aged (a) PTzBI-dF:L8BO, (b) PTzBI-dF:Y6 and (c) PTzBI-dF:L8BO:Y6 based devices.

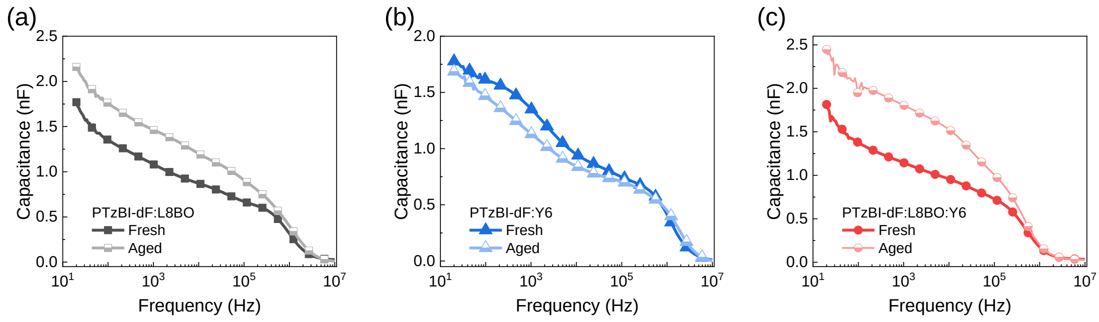

Supplementary Fig. 36 | *C-F*. Capacitance versus frequency at 0.5V applied bias for fresh and aged (a) PTzBI-dF:L8BO, (b) PTzBI-dF:Y6 and (c) PTzBI-dF:L8BO:Y6 based devices.

Supplementary Table 1 | Stability parameters of OSCs based on Y6 and its derivatives under different aging conditions reported by previous literatures.

Active layer	Device structures	Aging conditions	Aged time (h)	Best PCE (%)	Remain (%)
PM6:Y6[1]	ITO/PEDOT:PSS/active layer/PDINO/Ag	N_2, 140 ℃	1200	16.20	67
PM6:Y6[2]	ITO/ZnO/active layer/MoO_3/Ag	N_2, 100 ℃	600	15.4	62
PM6:Y6[3]	ITO/ZnO/active layer/MoO_3/Al	N_2, 55 ℃, LED (1 sun)	500	15.32	53
PM6:Y6[4]	ITO/ZnO/active layer/MoO_3/Ag	N_2, LED (1 sun)	600	15.96	77
PM6:Y6[5]	ITO/PEDOT:PSS/active layer/PDINO/Al	N_2	720	15.6	79.9
PM6:Y6[6]	ITO/PEDOT:PSS/active layer/DPO/Al	1 sun	225	15.2	44
PM6:Y6[7]	ITO/PEDOT:PSS/active layer/PDIN/Ag	N_2	2500	15.78	73
PM6:Y6[8]	ITO/ZnO/active layer/MoO_3/Al	maximum power point (mpp)	200	15.03	50
PM6:Y6[9]	ITO/PEDOT: PSS/active layer/PDINO/Al	1 sun	160	16.07	66
PM6:Y6[10]	ITO/PEDOT:PSS/active layer/PDINN/Ag	N_2	240	14.5	60

PM6:Y6[11]	ITO/PEDOT:PSS/active layer/PNDIT-F3N/Ag	AM 1.5 G, 100 mW cm^{-2}	74	16.70	77
PM6:BTP-4Cl[12]	ITO/PEDOT:PSS/active layer/PDIN/Al	N_2, 60 ℃	150	15.60	53
PM6:BTP-4Cl[13]	ITO/PEDOT:PSS/active layer/PDIN/Al	N_2	300	15.97	61.43
PM7:Y7[14]	ITO/ZnO/active layer/MoO_3/Ag	N_2, 80 ℃	500	15.13	78
PM6:BTP-eC9[15]	ITO/PEDOT:PSS/active layer/perylene-diimide/Ag	N_2, 85 ℃	240	17.18	63
D18-Cl:L8-BO[16]	ITO/PEDOT:PSS/active layer/PNDIT-F3N/Ag	AM 1.5 G, 100 mW cm^{-2}	24	15.1	88
D18-Cl:Y6[17]	ITO/PEDOT:PSS/active layer/PDINN/Ag	ambient, 80 ℃	2.5	16.99	69
D18-Cl:Y6[18]	ITO/PEDOT:PSS/active layer/PDINN/Ag	N_2	2500	17.73	92.04
PTQ10:Y6[19]	ITO/ZnO/active layer/MoO_3/Ag	N_2	1340	15.03	81
PM6:N3[8]	ITO/ZnO/active layer/MoO_3/Al	maximum power point (mpp)	200	15.00	50
PM6:Y6-BO[20]	ITO/ZnO/active layer/MoO_3/Ag	LED, 100 mW cm^{-2}	1000	15.1	13

Supplementary Table 2 | Fitting results of 100 and π–π stacking peaks of the neat and blend films.

Film	Sector	Type	Location [Å^{-1}]	d-spacing [Å]	FWHM [a] [Å^{-1}]	CCL [b] [Å]
PTzBI-dF	IP	Lorentzian	0.29	21.73	0.08	74.37
	OOP	Lorentzian	1.71	3.67	0.32	17.89
L8BO	IP	Lorentzian	0.42	14.92	0.15	37.93
	OOP	Lorentzian	1.72	3.65	0.39	14.64
L8BO:Y6	IP_0	Lorentzian	0.32	19.62	0.10	56.52
	IP_1	Lorentzian	0.42	14.95	0.09	62.11
	OOP	Lorentzian	1.74	3.61	0.31	18.06
Y6	IP_0	Lorentzian	0.29	21.58	0.09	66.49
	IP_1	Lorentzian	0.42	15.02	0.11	50.92
	OOP	Lorentzian	1.75	3.59	0.26	21.57

[a] FWHM is the full width at half maximum; [b] Crystalline coherence length (CCL) is estimated from Scherrer analysis: CCL = $2\pi K$/FWHM, where K is the shape factor (K = 0.9 here).

Supplementary Table 3 | Optical features obtained from the UV-vis absorption spectra of L8BO:Y6 blends with various ratios.

L8BO:Y6	Peak Index	Peak Type	Area Fit	Center Max	Max Height	FWHM
1.2:0	black	Gaussian	0.11	1.54	0.74	0.14
	red	Gaussian	0.13	1.70	0.52	0.24
	blue	Gaussian	0.03	1.95	0.16	0.19
0.9:0.3	black	Gaussian	0.11	1.53	0.73	0.15
	red	Gaussian	0.13	1.69	0.51	0.24
	blue	Gaussian	0.05	1.93	0.19	0.23
0.7:0.5	black	Gaussian	0.12	1.52	0.74	0.15
	red	Gaussian	0.14	1.69	0.53	0.25
	blue	Gaussian	0.05	1.93	0.20	0.22
0.5:0.7	black	Gaussian	0.11	1.51	0.71	0.15
	red	Gaussian	0.13	1.66	0.51	0.24
	blue	Gaussian	0.06	1.89	0.23	0.26
0.3:0.9	black	Gaussian	0.11	1.49	0.71	0.15
	red	Gaussian	0.12	1.64	0.50	0.23
	blue	Gaussian	0.08	1.87	0.26	0.29
0:1.2	black	Gaussian	0.11	1.48	0.73	0.14
	red	Gaussian	0.12	1.63	0.49	0.23
	blue	Gaussian	0.08	1.86	0.28	0.28

Supplementary Table 4 | The ions intensity obtained from neat films.

Neat films	Peak label	m/z	Intensity	$I_{(CNO-)}/I_{(CN-)}$
PTzBI-dF	CNO^-	42.0	8.43×10^5	2.26×10^{-1}
	CN^-	26.0	3.73×10^6	
L8BO	CNO^-	42.0	2.98×10^4	4.08×10^{-3}
	CN^-	26.0	7.30×10^6	
Y6	CNO^-	42.0	2.98×10^4	4.08×10^{-3}
	CN^-	26.0	7.30×10^6	

Supplementary Table 5 | Photovoltaic parameters of PTzBI-dF based OSCs under simulated AM 1.5G irradiation (100 mW cm^{-2}) and the hole and electron mobilities measured by SCLC method for PTzBI-dF:L8BO:Y6 systems.

PTzBI-dF:L8BO:Y6	V_{OC} (V)	J_{SC} (mA cm^{-2})	FF (%)	PCE (%)	μ_h (cm^2 V^{-1} s^{-1})	μ_e (cm^2 V^{-1} s^{-1})
1:1.2:0	0.882	24.51	76.49	16.74	4.81×10^{-4}	4.51×10^{-4}
1:0.9:0.3	0.876	26.00	77.18	17.58	5.56×10^{-4}	6.02×10^{-4}
1:0.7:0.5	0.860	26.95	78.78	18.26	6.18×10^{-4}	6.36×10^{-4}
1:0.5:0.7	0.856	26.89	74.10	17.07	5.49×10^{-4}	6.19×10^{-4}
1:0.3:0.9	0.842	26.60	73.46	16.45	5.35×10^{-4}	5.85×10^{-4}
1:0:1.2	0.829	26.94	72.66	16.23	4.89×10^{-4}	5.36×10^{-4}

Supplementary Table 6 | Fitting parameters for corresponding graphs.

Blend	n (V_{OC}-Plight)	α (J_{SC}-Plight)	R_s (Ω cm^2)	R_{sh} (Ω cm^2)
PTzBI-dF:L8BO	1.27 kT/q	0.95	1.11	3.17×10^5
PTzBI-dF:Y6	1.38 kT/q	0.91	1.12	7.62×10^4
PTzBI-dF:L8BO:Y6	1.17 kT/q	0.98	0.91	4.63×10^5

Supplementary Table 7 | Photovoltaic parameters of specific devices based on L8BO:Y6 with different blending ratios under simulated AM 1.5G irradiation (100 mW cm^{-2}).

L8BO:Y6	V_{OC} (V)	J_{SC} (mA cm^{-2})	FF (%)	PCE (%)
1.2:0	0.186	6.99×10^{-2}	24.68	3.2×10^{-3}
0.9:0.3	0.426	8.88×10^{-2}	26.02	9.8×10^{-3}
0.7:0.5	0.674	1.14×10^{-1}	24.00	1.83×10^{-2}
0.5:0.7	0.689	1.29×10^{-1}	24.04	2.13×10^{-2}
0.3:0.9	0.760	1.58×10^{-1}	23.78	2.86×10^{-2}
0:1.2	0.786	2.67×10^{-1}	24.18	5.07×10^{-2}

Supplementary Table 8 | Measured and calculated parameters to quantify the energy losses.

Blend	E_g [a)] (eV)	$qV_{OC,SQ}$ (eV)	ΔE_1 [b)] (eV)	$qV_{OC,rad}$ (eV)	ΔE_2 [c)] (eV)	EQE_{EL} (%)	ΔE_3 [d)] (eV)	V_{OC} cal (V)
PTzBI-dF:L8BO	1.43	1.16	0.27	1.10	0.06	1.22×10^{-2}	0.23	0.87
PTzBI-dF:Y6	1.39	1.13	0.26	1.07	0.06	6.10×10^{-3}	0.25	0.82
PTzBI-dF:L8BO:Y6	1.41	1.15	0.26	1.08	0.07	1.15×10^{-2}	0.23	0.85

[a)] The optical bandgaps (E_g) was extract from the derivative of EQE edges; [b)] $\Delta E_1 = E_g - qV_{rad,SQ}$; [c)] $\Delta E_2 = qV_{rad,SQ} - qV_{OC,rad}$; [d)] $\Delta E_3 = k_B T \ln EQE_{EL}$.

Supplementary Table 9 | Photovoltaic parameters of PTzBI-dF:L8BO, PTzBI-dF:Y6, PTzBI-dF:L8BO:Y6 inverted devices under simulated AM 1.5G irradiation (100 mW cm^{-2})

Active layer	V_{OC} (V)	J_{SC} (mA cm^{-2})	FF (%)	PCE (%)
PTzBI-dF:L8BO	0.897	23.36	72.29	15.15
PTzBI-dF:Y6	0.840	25.25	70.72	14.92
PTzBI-dF:L8BO:Y6	0.876	25.12	73.83	16.25

Supplementary Table 10 | Parameters of energy levels and optical bandgap

Acceptors	E_{ox}[a] (V)	HOMO (eV)	E_{re}[b] (V)	LUMO (eV)	λ_{onset} (nm)	$E_{gap, optical}$[c] (eV)
L8BO	1.33	-5.73	-0.53	-3.87	836.9	1.48
Y6	1.28	-5.68	-0.50	-3.90	874.5	1.42
L8BO:Y6	1.30	-5.70	-0.51	-3.89	854.0	1.45

[a] $E_{HOMO} = -e[E_{ox} - E_{Fc/Fc+} + 4.8]$ eV; [b] $E_{LUMO} = -e[E_{re} - E_{Fc/Fc+} + 4.8]$ eV; [c] Optical bandgap extracted from absorption spectra.

Supplementary Table 11 | Photovoltaic parameters of D18 and PM6 separately combined with L8BO, Y6 and L8BO:Y6 as active layers-based OSCs under simulated AM 1.5G irradiation (100 mW cm^{-2})

Active layers	V_{OC} (V)	J_{SC} (mA cm^{-2})	FF (%)	PCE (%)
D18:L8BO	0.885	24.75	65.57	14.47
D18:Y6	0.827	25.49	69.60	14.80
D18:L8BO:Y6	0.854	24.71	72.15	15.35
PM6:L8BO	0.867	25.20	68.13	14.89
PM6:Y6	0.814	26.24	69.04	14.76
PM6:L8BO:Y6	0.855	25.39	68.40	14.97

Supplementary Table 12 | Fitting results of 100 and π–π stacking peaks for fresh and aged PTzBI-dF:L8BO:Y6 with various L8BO:Y6 ratios

PTzBI-dF: L8BO:Y6	Sector	Type	Location [$Å^{-1}$]	d-spacing [Å]	FWHM [a] [$Å^{-1}$]	CCL [b] [Å]
1:1.2:0_Fresh	IP	Lorentzian	0.31	20.06	0.11	49.36
	OOP	Lorentzian	1.73	3.62	0.36	15.63
1:1.2:0_Aged	IP	Lorentzian	0.31	20.07	0.11	49.79
	OOP	Lorentzian	1.73	3.62	0.35	15.97
1:0.9:0.3_Fresh	IP	Lorentzian	0.36	17.42	0.11	53.02
	OOP	Lorentzian	1.73	3.62	0.33	17.22
1:0.9:0.3_Aged	IP	Lorentzian	0.34	18.68	0.11	53.04
	OOP	Lorentzian	1.73	3.63	0.30	18.54
1:0.7:0.5_Fresh	IP	Lorentzian	0.30	20.84	0.10	55.91
	OOP	Lorentzian	1.73	3.62	0.32	17.45
1:0.7:0.5_Aged	IP	Lorentzian	0.33	18.94	0.10	54.16
	OOP	Lorentzian	1.72	3.64	0.31	17.95
1:0.5:0.7_Fresh	IP	Lorentzian	0.30	21.18	0.10	57.14
	OOP	Lorentzian	1.73	3.63	0.32	17.63
1:0.5:0.7_Aged	IP	Lorentzian	0.33	19.28	0.09	59.68
	OOP	Lorentzian	1.73	3.63	0.30	18.98
1:0.3:0.9_Fresh	IP	Lorentzian	0.29	21.44	0.09	63.24
	OOP	Lorentzian	1.73	3.62	0.32	17.89
1:0.3:0.9_Aged	IP	Lorentzian	0.32	19.36	0.09	60.85
	OOP	Lorentzian	1.74	3.61	0.30	18.65
1:0:1.2_Fresh	IP	Lorentzian	0.29	21.82	0.08	69.60
	OOP	Lorentzian	1.74	3.62	0.30	18.62
1:0:1.2_Aged	IP	Lorentzian	0.32	19.50	0.09	65.82
	OOP	Lorentzian	1.74	3.61	0.29	19.32

[a] FWHM is the full width at half maximum; [b] Crystalline coherence length (CCL) is estimated from Scherrer analysis: CCL = $2\pi K/\text{FWHM}$, where K is the shape factor (K = 0.9 here).

Supplementary Table 13 | Optical features obtained from the fresh and thermal aged L8BO, Y6 and L8BO:Y6 films of absorption spectra decomposition.

Films	Peak Index	Peak Type	Area Fit	Center Max	Max Height	FWHM
L8BO_Fresh	black	Gaussian	0.10	1.55	0.65	0.14
	red	Gaussian	0.12	1.71	0.47	0.24
	blue	Gaussian	0.04	1.95	0.17	0.21
L8BO_Aged	black	Gaussian	0.09	1.53	0.66	0.13
	red	Gaussian	0.12	1.69	0.48	0.23
	blue	Gaussian	0.04	1.94	0.15	0.22
Y6_Fresh	black	Gaussian	0.07	1.48	0.47	0.14
	red	Gaussian	0.08	1.63	0.33	0.24
	blue	Gaussian	0.05	1.86	0.18	0.27
Y6_Aged	black	Gaussian	0.08	1.48	0.51	0.14
	red	Gaussian	0.09	1.63	0.36	0.23
	blue	Gaussian	0.06	1.86	0.21	0.28
L8BO:Y6_Fresh	black	Gaussian	0.08	1.52	0.48	0.15
	red	Gaussian	0.09	1.68	0.34	0.24
	blue	Gaussian	0.03	1.92	0.13	0.24
L8BO:Y6_Aged	black	Gaussian	0.09	1.52	0.55	0.15
	red	Gaussian	0.10	1.68	0.40	0.24
	blue	Gaussian	0.04	1.93	0.17	0.23

Supplementary Table 14 | Optical features obtained from the fresh and thermal aged L8BO, Y6 and L8BO:Y6 films of PL spectral decomposition.

Films	Peak Index	Peak Type	Area Fit	Center Max	Max Height	FWHM
L8BO_Fresh	black	Lorentzian	0.10	1.19	0.43	0.14
	red	Lorentzian	0.16	1.26	0.79	0.13
	blue	Lorentzian	0.02	1.36	0.18	0.07
L8BO_Aged	black	Lorentzian	0.12	1.19	0.52	0.15
	red	Lorentzian	0.15	1.25	0.72	0.13
	blue	Lorentzian	0.02	1.36	0.18	0.06
Y6_Fresh	black	Lorentzian	0.12	1.16	0.61	0.12
	red	Lorentzian	0.11	1.23	0.61	0.11
	blue	Lorentzian	0.08	1.29	0.56	0.10
Y6_Aged	black	Lorentzian	0.13	1.15	0.69	0.12
	red	Lorentzian	0.10	1.22	0.57	0.11
	blue	Lorentzian	0.08	1.28	0.50	0.11
L8BO:Y6_Fresh	black	Lorentzian	0.11	1.17	0.53	0.13
	red	Lorentzian	0.13	1.24	0.71	0.12
	blue	Lorentzian	0.06	1.31	0.34	0.10
L8BO:Y6_Aged	black	Lorentzian	0.11	1.16	0.52	0.13
	red	Lorentzian	0.13	1.23	0.68	0.12
	blue	Lorentzian	0.07	1.30	0.38	0.11

Supplementary Table 15 | Optical features obtained from the fresh and thermal aged PTzBI-dF:L8BO, PTzBI-dF:Y6 and PTzBI-dF: L8BO:Y6 films of absorption spectra decomposition.

Films	Peak Index	Peak Type	Area Fit	Center Max	Max Height	FWHM
PTzBI-dF:L8BO_Fresh	black	Gaussian	0.05	1.54	0.48	0.09
	red	Gaussian	0.06	1.65	0.36	0.17
	blue	Gaussian	0.18	1.97	0.46	0.37
	green	Gaussian	0.05	2.36	0.12	0.42
PTzBI-dF:L8BO_Aged	black	Gaussian	0.05	1.53	0.54	0.09
	red	Gaussian	0.07	1.64	0.39	0.17
	blue	Gaussian	0.19	1.97	0.49	0.37
	green	Gaussian	0.06	2.37	0.13	0.44
PTzBI-dF:Y6_Fresh	black	Gaussian	0.03	1.50	0.30	0.09
	red	Gaussian	0.04	1.60	0.25	0.16
	blue	Gaussian	0.08	1.93	0.29	0.26
	green	Gaussian	0.11	2.12	0.24	0.44
PTzBI-dF:Y6_Aged	black	Gaussian	0.03	1.50	0.27	0.10
	red	Gaussian	0.04	1.61	0.22	0.17
	blue	Gaussian	0.08	1.94	0.26	0.27
	green	Gaussian	0.09	2.12	0.20	0.44
PTzBI-dF: L8BO:Y6_Fresh	black	Gaussian	0.04	1.53	0.36	0.09
	red	Gaussian	0.05	1.62	0.32	0.16
	blue	Gaussian	0.17	1.96	0.46	0.35
	green	Gaussian	0.07	2.26	0.14	0.48
PTzBI-dF: L8BO:Y6_Aged	black	Gaussian	0.04	1.52	0.35	0.09
	red	Gaussian	0.05	1.62	0.32	0.16
	blue	Gaussian	0.18	1.97	0.46	0.38
	green	Gaussian	0.07	2.34	0.13	0.48

Supplementary Table 16 | Optical features obtained from the fresh and thermal aged PTzBI-dF:L8BO, PTzBI-dF:Y6 and PTzBI-dF: L8BO:Y6 films of PL spectral decomposition.

Films	Peak Index	Peak Type	Area Fit	Center Max	Max Height	FWHM
PTzBI-dF:L8BO_Fresh	black	Lorentzian	0.21	1.21	0.67	0.20
	red	Lorentzian	0.09	1.27	0.54	0.11
	blue	Lorentzian	0.02	1.35	0.19	0.06
PTzBI-dF:L8BO_Aged	black	Lorentzian	0.22	1.21	0.65	0.21
	red	Lorentzian	0.09	1.27	0.50	0.12
	blue	Lorentzian	0.03	1.34	0.25	0.08
PTzBI-dF:Y6_Fresh	black	Lorentzian	0.26	1.16	0.93	0.18
	red	Lorentzian	0.11	1.27	0.68	0.10
	blue	Lorentzian	0.02	1.33	0.23	0.06
PTzBI-dF:Y6_Aged	black	Lorentzian	0.25	1.16	0.94	0.17
	red	Lorentzian	0.07	1.25	0.48	0.10
	blue	Lorentzian	0.04	1.30	0.37	0.07
PTzBI-dF: L8BO:Y6_Fresh	black	Lorentzian	0.21	1.17	0.73	0.18
	red	Lorentzian	0.11	1.27	0.65	0.11
	blue	Lorentzian	0.03	1.33	0.26	0.07
PTzBI-dF: L8BO:Y6_Aged	black	Lorentzian	0.22	1.17	0.75	0.19
	red	Lorentzian	0.11	1.27	0.65	0.11
	blue	Lorentzian	0.03	1.33	0.28	0.07

Supplementary Table 17 | Optical features obtained from the decomposition of UV/Vis and PL spectra

Films	Condition	Φ_{RET} / Φ_{PL}
L8BO	Fresh	0.56
	Aged	0.52
Y6	Fresh	0.17
	Aged	0.14
L8BO:Y6	Fresh	0.22
	Aged	0.15
PTzBI-dF:L8BO	Fresh	0.39
	Aged	0.19
PTzBI-dF:Y6	Fresh	0.34
	Aged	0.04
PTzBI-dF:L8BO:Y6	Fresh	0.40
	Aged	0.36

Supplementary Table 18 | Fitting parameters for corresponding graphs.

Blends	Condition	R_0 (Ω cm^2)	R_1 (kΩ cm^2)	P (E,T) (%)	τ_{TPC} (ns)	τ_{TPV} (μs)
PTzBI-dF:L8BO	Fresh	13.21	438.39	97.25	69	8.45
	Aged	27.52	386.82	92.19	102	6.05
PTzBI-dF:Y6	Fresh	13.36	384.31	93.87	81	7.50
	Aged	26.48	286.77	87.48	186	4.95
PTzBI-dF: L8BO:Y6	Fresh	11.70	526.28	98.20	28	11.4
	Aged	14.87	514.96	97.66	36	9.85

Supplementary Table 19 | Fitting parameters for corresponding graphs

Blends	Slope (F^{-2} V^{-1})	N_A (cm^{-3})	V_{bi} (V)	E_t (eV)	σ (eV)	N_t (cm^{-3})
PTzBI-dF:L8BO _Fresh	-4.11×10^{17}	4.30×10^{16}	0.98	0.34	0.04	6.45×10^{15}
PTzBI-dF:L8BO_ Aged	-3.45×10^{17}	5.12×10^{16}	1.46	0.33	0.05	9.85×10^{15}
PTzBI-dF:Y6_ Fresh	-4.01×10^{17}	4.41×10^{16}	0.85	0.33	0.05	9.75×10^{15}
PTzBI-dF:Y6_ Aged	-3.21×10^{17}	5.51×10^{16}	1.25	0.33	0.05	1.16×10^{16}
PTzBI-dF: L8BO:Y6_Fresh	-6.20×10^{17}	2.85×10^{16}	1.00	0.36	0.05	4.09×10^{15}
PTzBI-dF: L8BO:Y6_Aged	-6.00×10^{17}	2.95×10^{16}	1.07	0.36	0.05	5.35×10^{15}

Supplementary References

1 Zhang L, et al. Enhanced efficiency and excellent thermostability in organic photovoltaics via ternary strategy with twisted conjugated compound. *Small* **17**, 2103537 (2021).

2 Chang B, et al. Incorporating indium selenide nanosheets into a polymer/small molecule binary blend active layer enhances the long-term stability and performance of its organic photovoltaics. *ACS Appl. Mater. Interfaces* **12**, 55023-55032 (2020).

3 Wei Y, Liang N, Jiang W, Zhai T, Wang Z. Rylene-fullerene hybrid an emerging electron acceptor for high-performing and photothermal-stable ternary solar cells. *Small* **18**, 2104060 (2022).

4 Cheng Y, et al. Oligomer-assisted photoactive layers enable >18 % efficiency of organic solar cells. *Angew. Chem. Int. Ed.* **61**, 202200329 (2022).

5 You W, et al. Adjusting the active layer morphology via an amorphous acceptor solid additive for efficient and stable non fullerene organic solar cells. *Solar RRL* **5**, 2100532 (2021).

6 Gasparini N, et al. Exploiting ternary blends for improved photostability in high-efficiency organic solar cells. *ACS Energy Lett.* **5**, 1371-1379 (2020).

7 Chen J, et al. Layer-by-layer processed pm6:Y6-based stable ternary organic solar cells with improved efficiency over 18% by incorporating an asymmetric thieno[3,2-b]indole-based acceptor. *Adv. Funct. Mater.* **32**, 2200629 (2022).

8 Liu B, et al. Simultaneously achieving highly efficient and stable polymer:Non-fullerene solar cells enabled by molecular structure optimization and surface passivation. *Adv. Sci.* **9**, 2104588 (2022).

9 Zhong L, et al. Naphthalene as a thermal-annealing-free volatile solid additive in non-fullerene organic solar cells with improved performance and reproducibility. *Adv. Funct. Mater.* **32**, 2201080 (2022).

10 Song X, et al. Process-aid solid engineering triggers delicately modulation of y-series non-fullerene acceptor for efficient organic solar cells. *Adv. Mater.* **34**, 2200907 (2022).

11 Xie Y, et al. High-efficiency organic solar cells enabled by an alcohol-washable solid additive. *Sci. China Chem.* **64**, 2161-2168 (2021).

12 Zhang K-N, et al. Exploring the mechanisms of exciton diffusion improvement in ternary polymer solar cells: From ultrafast to ultraslow temporal scale. *Nano Energy* **79**, 105513 (2021).

13 Zhang KN, et al. Suppressing kinetic aggregation of non-fullerene acceptor via versatile alloy states enables high-efficiency and stable ternary organic solar cells. *Adv. Funct. Mater.* **31**, 2100316 (2021).

14 Gokulnath T, et al. A wide-bandgap π-conjugated polymer for high-performance ternary organic solar cells with an efficiency of 17.40%. *Nano Energy* **89**, 106323 (2021).

15 Zhang KN, et al. Reducing limitations of aggregation-induced photocarrier trapping for photovoltaic stability via tailoring intermolecular electron–phonon coupling in highly efficient quaternary organic solar cells. *Adv. Energy Mater.* **12**, 2103371 (2021).

16 Cai G, et al. Pushing the efficiency of high open-circuit voltage binary organic solar cells by vertical morphology tuning. *Adv. Sci.* **9**, 2200578 (2022).

17 Li M, et al. Non-fullerene acceptor alloy strategy enabling stable ternary polymer solar cells with efficiency of 17.74%. *J. Mater. Chem. C* **10**, 3207-3216 (2022).

18 Li X, et al. High-efficiency sequential-cast organic solar cells enabled by dual solvent-controlled polymer aggregation. *Solar RRL* **6**, 2200076 (2022).

19 Qiu B, et al. Understanding the effect of the third component pc 71 bm on nanoscale morphology and photovoltaic properties of ternary organic solar cells. *Solar RRL* **4**, 1900540 (2020).

20 Zhang X, et al. Simultaneously enhanced efficiency and operational stability of non-fullerene organic solar cells via solid-additive-mediated aggregation control. *Small* **17**, 2102558 (2021).
