# Supplementary material for: Mastering morphology of non-fullerene acceptors towards long-term stable organic solar cells
Source: Nat Commun. 2023 May 10;14:2688. doi: 10.1038/s41467-023-38306-x (PMC10172308; doi:10.1038/s41467-023-38306-x)
Supplement: Supplementary file 3 — Solar Cells Reporting Summary [file 41467_2023_38306_MOESM3_ESM.pdf]

nature research

Corresponding author(s): Ning Li, Fei Huang, Lei Ying

Solar Cells Reporting Summary

Nature Research wishes to improve the reproducibility of the work that we publish. This form is intended for publication with all accepted papers reporting the characterization of photovoltaic devices and provides structure for consistency and transparency in reporting. Some list items might not apply to an individual manuscript, but all fields must be completed for clarity.

For further information on Nature Research policies, including our data availability policy, see Authors & Referees.

Experimental design

Please check: are the following details reported in the manuscript?

1. Dimensions

Item	Yes	No	Details
Area of the tested solar cells	☒	☐	Section "Device fabrication".
Method used to determine the device area	☒	☐	Section "Device fabrication".

2. Current-voltage characterization

Item	Yes	No	Details
Current density-voltage (J-V) plots in both forward and backward direction	☐	☒	Generally, organic solar cell devices do not have the hysteresis problem. The current-voltage (J-V) plots in forward direction are supplied in Figure 1f and Supplementary Figure 6.
Voltage scan conditions<br>*For instance: scan direction, speed, dwell times*	☒	☐	Section "Instruments and characterizations".
Test environment<br>*For instance: characterization temperature, in air or in glove box*	☒	☐	Our devices were characterized at room temperature in a glove box.
Protocol for preconditioning of the device before its characterization	☐	☒	No precondition before its characterization
Stability of the J-V characteristic<br>*Verified with time evolution of the maximum power point or with the photocurrent at maximum power point; see ref. 7 for details.*	☒	☐	We tested the long-term stability at elevated temperatures

3. Hysteresis or any other unusual behaviour

Item	Yes	No	Details
Description of the unusual behaviour observed during the characterization	☐	☒	No hysteresis or other unusual behavior was observed during the characterization of the solar cells. In general, organic solar cells do not have hysteresis problems.
Related experimental data	☐	☒	No hysteresis or other unusual behavior was observed during the characterization of the solar cells.

4. Efficiency

Item	Yes	No	Details
External quantum efficiency (EQE) or incident photons to current efficiency (IPCE)	☒	☐	Figure 1g.
A comparison between the integrated response under the standard reference spectrum and the response measure under the simulator	☒	☐	Table 1.
For tandem solar cells, the bias illumination and bias voltage used for each subcell	☐	☒	We didn't fabricate tandem solar cells in this work

5. Calibration

Item	Yes	No	Details
Light source and reference cell or sensor used for the characterization	☒	☐	Section "Instruments and characterizations".
Confirmation that the reference cell was calibrated and certified	☒	☐	Section "Instruments and characterizations".

Calculation of spectral mismatch between the reference cell and the devices under test

☐ Yes
☒ No

The spectral mismatch factor was determined by the manufacturer of the light source and the corresponding engineer. We do not have the detailed information for the specific factor.

6. Mask/aperture

Size of the mask/aperture used during testing

☒ Yes
☐ No

The size of aperture used in this work is 0.04-cm2 .

Variation of the measured short-circuit current density with the mask/aperture area

☐ Yes
☒ No

We only used the same aperture for characterization in this work

7. Performance certification

Identity of the independent certification laboratory that confirmed the photovoltaic performance

☐ Yes
☒ No

We didn't certify the efficiency of our OSCs, because this work mainly focuses on the thermal stability of OSCs, and does not report a champion efficiency.

A copy of any certificate(s)
Provide in Supplementary Information

☐ Yes
☒ No

No certification.

8. Statistics

Number of solar cells tested

☒ Yes
☐ No

We tested at least 10 cells for each type of devices.

Statistical analysis of the device performance

☒ Yes
☐ No

Statistical results of the devices are listed in Table 1.

9. Long-term stability analysis

Type of analysis, bias conditions and environmental conditions
For instance: illumination type, temperature, atmosphere humidity, encapsulation method, preconditioning temperature

☒ Yes
☐ No

Section "Thermal stability of OSCs and film morphology".
